# Ranking the synthesizability of hypothetical zeolites with the sorting hat†

**DOI:** 10.1039/d2dd00056c

**Published:** 2022-10-12

**Authors:** Benjamin A. Helfrecht, Giovanni Pireddu, Rocio Semino, Scott M. Auerbach, Michele Ceriotti

**Affiliations:** Laboratory of Computational Science and Modeling, Institut des Matériaux, École Polytechnique Fédérale de Lausanne 1015 Lausanne Switzerland; PASTEUR, Département de Chimie, École Normale Supérieure, PSL University, Sorbonne Université, CNRS 24 rue Lhomond 75005 Paris France; Sorbonne Université, CNRS, Physico-chimie des Electrolytes et Nanosystèmes Interfaciaux PHENIX F-75005 Paris France rocio.semino@sorbonne-universite.fr; Department of Chemistry and Department of Chemical Engineering, University of Massachusetts Amherst Amherst MA 01003 USA auerbach@umass.edu; Laboratory of Computational Science and Modeling, Institut des Matériaux, École Polytechnique Fédérale de Lausanne 1015 Lausanne Switzerland michele.ceriotti@epfl.ch; ICGM, Univ. Montpellier, CNRS, ENSCM Montpellier France

## Abstract

Zeolites are nanoporous alumino-silicate frameworks widely used as catalysts and adsorbents. Even though millions of siliceous networks can be generated by computer-aided searches, no new hypothetical framework has yet been synthesized. The needle-in-a-haystack problem of finding promising candidates among large databases of predicted structures has intrigued materials scientists for decades; yet, most work to date on the zeolite problem has been limited to intuitive structural descriptors. Here, we tackle this problem through a rigorous data science scheme—the “Zeolite Sorting Hat”—that exploits interatomic correlations to discriminate between real and hypothetical zeolites and to partition real zeolites into compositional classes that guide synthetic strategies for a given hypothetical framework. We find that, regardless of the structural descriptor used by the Zeolite Sorting Hat, there remain hypothetical frameworks that are incorrectly classified as real ones, suggesting that they might be good candidates for synthesis. We seek to minimize the number of such misclassified frameworks by using as complete a structural descriptor as possible, thus focusing on truly viable synthetic targets, while discovering structural features that distinguish real and hypothetical frameworks as an output of the Zeolite Sorting Hat. Further ranking of the candidates can be achieved based on thermodynamic stability and/or their suitability for the desired applications. Based on this workflow, we propose three hypothetical frameworks differing in their molar volume range as the top targets for synthesis, each with a composition suggested by the Zeolite Sorting Hat. Finally, we analyze the behavior of the Zeolite Sorting Hat with a hierarchy of structural descriptors including intuitive descriptors reported in previous studies, finding that intuitive descriptors produce significantly more misclassified hypothetical frameworks, and that more rigorous interatomic correlations point to second-neighbor Si–O distances around 3.2–3.4 Å as the key discriminatory factor.

## Abstract 2

The Zeolite Sorting Hat: a data-driven framework that identifies hypothetical silica frameworks that are promising synthetic targets, and suggests the most likely compositional class for the candidate materials.

## Graphical abstract

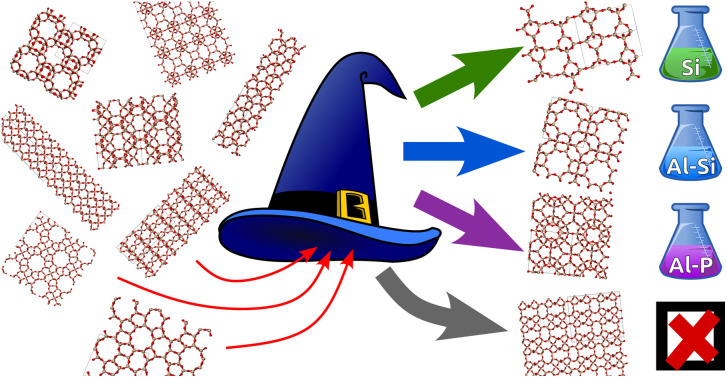

## 1 Introduction

Zeolites are nanoporous crystalline materials with exceptionally high thermal and hydrothermal stabilities, making them excellent candidates for a range of present and future technologies based on shape selectivity. Because of their controlled nanoporosity and acidic properties, zeolites find application in a myriad of industrially relevant processes, predominantly in separation and catalysis.^1–3^ To accelerate zeolite discovery, databases of hypothetical zeolites have been created^4–7^ containing millions of new framework structures. These databases have been successfully screened identifying materials with desirable properties,^8–11^ but to date, none of them has been synthesized in the lab, a phenomenon referred to as the “zeolite conundrum”.^12^ A common assumption in the search for hypothetical zeolites that may be good candidates for synthesis is that the synthesizability of a given framework is correlated with its structural similarity to zeolites that have already been made. We note that prior work studying drug-like molecules relied on a similar principle of assessing synthesizability *via* the comparison of molecular fragments and computation of a synthesizability score.^13,14^

The great importance and challenge in fabricating new zeolites prompt several pressing questions: How do collections of real and hypothetical zeolites relate to each other in terms of structural diversity? Which structural features play the biggest role in distinguishing real and hypothetical zeolites? Which hypothetical zeolites are most likely synthesizable, and in which chemical composition? Previous attempts to answer these questions^15–22^ have relied on intuitive guesses for structural descriptors such as rings and angles. These rely on prior assumptions on the most relevant features and cannot be made systematically more complete,^23^ which hinders the construction of systematically-improvable classifiers. In the present work, we answer all these questions *via* rigorous data science methods combining unsupervised and supervised machine learning,^24^ along with the generalized convex hull (GCH) description of thermodynamic stability,^25^ yielding a new and powerful approach for sorting real^26^ and hypothetical^4–7^ zeolites, as well as finding promising zeolite candidates and suggesting likely chemical compositions for them.

## 2 Results

The scale of the zeolite conundrum can be appreciated by comparing the number of hypothetical frameworks with that of “real” zeolites. Different studies have suggested over 2 600 000 distinct topologies,^7^ and even the subset we consider here, which only contains particularly stable, fully connected frameworks selected among a larger pool of candidates, contains more than 300 000 all-silica structures (*i.e.*, the database of Deem and coworkers, henceforth denoted as DEEM; see ref. 7). In contrast, only 255 framework topologies have been collected in the International Zeolite Association database (henceforth denoted IZA), which can be realized in different compositional variations. To ensure that our comparisons are made on an equal footing, we perform our study on all-silica models. The great imbalance between known and hypothetical frameworks calls for a balancing act when applying data-driven analyses: models and structural descriptors must be flexible and sensitive enough to detect structural differences among all of the DEEM frameworks, but sufficiently robust and concise to extract useful information from a few hundred IZA entries without overfitting the smaller dataset.

To this end, we describe framework structures using the Smooth Overlap of Atomic Postions (SOAP) method,^27^ which allows systematic convergence of structural information by increasing the SOAP length scale and the order of atomic correlations (*e.g.*, distances, angles, and dihedrals^28,29^). In previous work, we proved this convergence by applying SOAP to machine-learn the framework density and lattice energy of DEEM frameworks, and showed that several heuristic fingerprints that have been used previously to analyze zeolite structures do not allow such convergence.^23^ Here we find that the DEEM-trained models accurately predict the same properties for IZA frameworks (see Table S1,† energy error below 0.20 kJ mol^−1^ Si). The accuracy of DEEM-trained predictions on IZA indicates substantial structural overlap between the two datasets, underscoring the significant challenge of telling them apart.

**Fig. 1:**
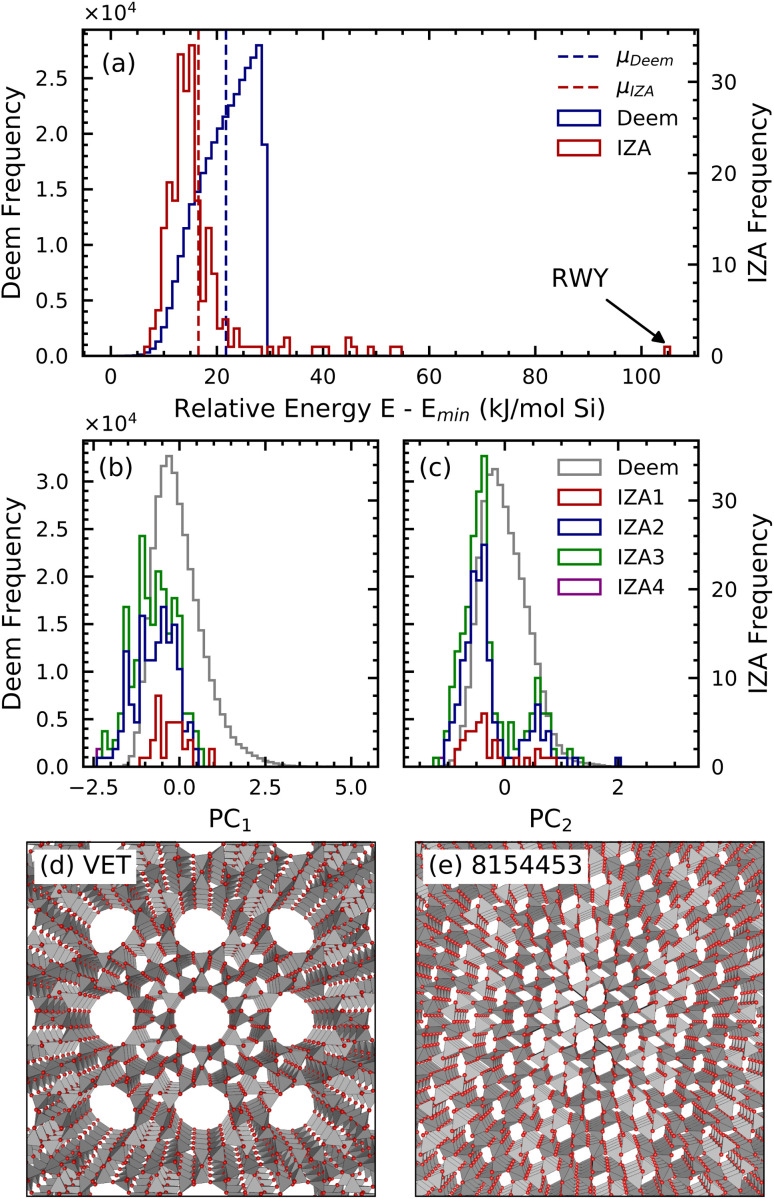
Histograms of (a) energies computed for the IZA and DEEM frameworks with GULP and of (b and c) values of the first two principal components of the power spectrum SOAP vectors of a subset of 10 000 DEEM frameworks and all 230 IZA frameworks. The histogram makes evident that the IZA frameworks are concentrated near the edge of the structural space defined by the DEEM frameworks. The PCA projection is defined only by the 10 000 DEEM frameworks. (d) Atomic snapshot of VET, the IZA framework based with the lattice energy closest to the IZA average. (e) Atomic snapshot of framework 8154453, the DEEM structure with the lattice energy closest to the DEEM average.

Armed with SOAP as a structural descriptor, we seek a method for discriminating IZA and DEEM entries. We note that classifiers based on lattice energy (Fig. 1(a)) or clustering along descriptors obtained by unsupervised dimensionality reduction (Fig. 1(b) and (c)) are both found to fail at telling IZA and DEEM apart (also see the point-clouds in Fig. S3†). Manual inspection of the structure of individual frameworks (two representative cases being shown in Fig. 1(d) and (e)) does not reveal any obvious discriminating feature, and is impractical given the size of DEEM. To solve this puzzle, we apply supervised learning to classifying zeolites, and denote our approach the “Zeolite Sorting Hat.”

In practice, however, we actually seek to solve an even harder problem: distinguishing subclasses of IZA based on composition to provide a rough starting point for viable synthesis routes for analogous DEEM frameworks. To do this, we parse IZA into subclasses (or “houses”) based on reference compositions, *i.e.*, the chemical composition of the material that first allowed the establishment of the framework topology, according to the IZA structure commission. This choice makes the assignment unique and future-proof, and is also consistent with the reasonable assumption that the reference material results from a robust synthetic approach. We reiterate that the sorting hat classification studied herein is based on geometry-optimized all-silica structures, and does not explicitly incorporate information on the composition. We nonetheless pursue a composition-based classification to determine the extent to which network structure predicts reference composition. Success in classifying frameworks according to their reference compositions can be benchmarked on IZA structures, and will test our hypothesis that framework structures can be used to infer information on synthesizability and on viable synthetic pathways. By extension, applying the trained compositional classifier to DEEM frameworks may suggest the chemical composition that should be pursued in the laboratory for a candidate structure, making our predictions of synthesizability more directly useful for materials chemists.

Analysis of the IZA database yields the following four houses: zeolite topologies with a pure-silica reference composition are assigned to IZA1; topologies whose reference composition contains O, but no Si, are classified as IZA3; topologies referenced to an intermediate fraction of Si (*e.g.*, aluminosilicates) are labeled as IZA2; and a single exotic framework (RWY) containing neither Si nor O is classified as IZA4 and is discarded from the present analysis as a structural and energetic outlier (see Fig. 1(a)). From a certain perspective, the distinction between pure-silica zeolites and alumino-silicate zeolites can be viewed as “shades of gray” rather than a qualitative difference warranting distinct classes. This perspective is supported in part by post-synthetic modifications such as de-alumination that can vary framework Al content levels. However, there is an alternative perspective – informed by direct zeolite synthesis – in which alumino-silicate zeolites and pure-silica zeolites should be seen as qualitatively different. Indeed, the zeolite synthesis methods developed in the 1950s by Barrer and coworkers^30^ use alkaline media to simulate geological processes at high pH to make alumino-silicates. In contrast, the methods pioneered by Edith Flanigen and coworkers in the 1970s^31^ apply fluoride-media at neutral pH to make pure silica zeolites. The focus of our work is informed by the direct-synthesis perspective because one cannot de-aluminate what one cannot make in the first place. As such, we see the virtue of distinguishing pure-silica zeolites (IZA1) and alumino-silicate zeolites (IZA2) as separate classes.

Through the lens of the principal components of the SOAP features shown in Fig. 1(b) and (c), these IZA houses occupy the same region in SOAP vector space, thus appearing indistinguishable according to the first two principal component directions. In summary, the various framework classes (DEEM/IZA1/IZA2/IZA3) cannot be effectively discriminated using an energetic criterion or through a simple unsupervised learning technique like PCA. In Section 2 we pursue such a compositional classification using a combined energy/density criterion for comparison with the rigorous results from SOAP. We have designed the Zeolite Sorting Hat to be robust, accurate, and interpretable. Together with SOAP representations as inputs (which provide a flexible, but physically sound description in terms of three-body correlations of the atom density) we use a linear support vector machine (SVM) whose outputs are decision functions: one function in the case of the two-class sort (DEEM/IZA) and four functions for the four-class sort (DEEM/IZA1/IZA2/IZA3). The use of a linear model simplifies the interpretation of results, and allows for interesting comparisons with classifications based on heuristic descriptors, which we show below in Fig. 5. Because of the scarcity of IZA structures relative to the size of the DEEM database, we implemented a class-balanced classification scheme that magnifies the weight of misclassified IZA structures in the training step.

The choice of an SVM classifier in which DEEM structures are considered non-synthesizable deserves some further comment. The problem of identifying synthesizable structures within DEEM falls squarely within the scope of positive-unlabeled (PU) methods, which use information on a set of inputs which are labeled (the IZA structures, which are known to be synthesizable) and a set of inputs whose classification is unknown (those from DEEM, which may be synthesizable or not). Application of PU methods^32^ requires knowing (or assuming) much about the relationship between the labeled and unlabeled datasets. In view of the empirical fact that no zeolite has been synthesized based on a hypothetical framework, it is reasonable to assume that only a tiny fraction of DEEM structures are amenable to synthesis, which led us to simply consider the hypothetical dataset as negative samples. This is analogous to the case of decoy molecules used in ligand binding studies,^33^ which are assumed to be negative samples even in the absence of data concerning their biological activity. Furthermore, in the most commonly-adopted setting (which assumes that labeled samples are selected completely at random from the set of positive samples) PU classification reduces to the application of classical binary classifiers, treating the unlabeled inputs as negative samples, but interpreting differently the resulting classification and the performance of the model.

It is also worth considering that—even though we formulate the classification problem in terms of clear-cut target classes—reality is more nuanced. The synthesis of a given framework could be facile, or extremely challenging. A framework could be made as pure SiO_2_, but also in different compositions. To recover such nuance, we will base most of our analysis on the magnitude of the decision functions that are used in the construction of the SVM, allowing us to rank the “IZA-ness” of a set of DEEM structures. Indeed, the SVM decision function goes beyond a binary classification by giving a degree of resemblance between two classes. To recover a binary classification, *i.e.*, to classify a particular structure as real or hypothetical, a decision boundary is defined. The decision boundary is a cutoff value that separates the otherwise continuous space, dividing the decision function into “IZA-like” and “DEEM-like” realms. To decide whether a structure is classified as IZA or DEEM, the Zeolite Sorting Hat computes the decision function for a particular zeolite and checks in which realm it falls (on which side of the decision boundary it lies). The location of the decision boundary is then varied to minimize false positives and false negatives in the classification, which provides a rough metric to assess the performance of the SVM. The Zeolite Sorting Hat was trained on a random half of IZA, and approximately 3% of DEEM (see Methods and the ESI† for details), and the results we report here refer to predictions made on the remainder of the datasets.

**Fig. 2:**
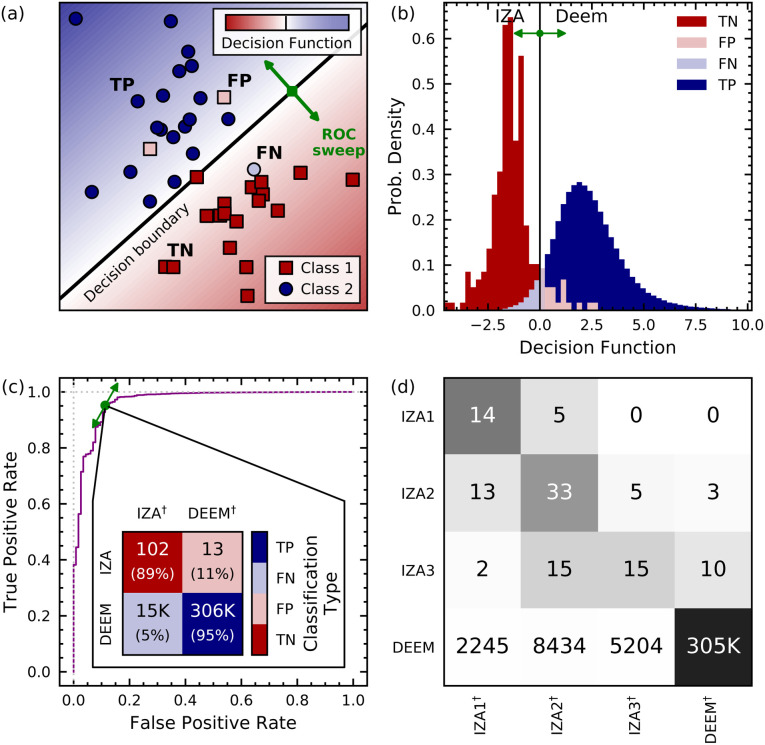
(a) Schematic of a support vector machine (SVM); each dot or square represents the feature vector for a given data point, the shading represents the value of the decision function, and the decision boundary location can be adjusted to optimize the classification (represented by a green arrow). (b) Histogram of decision function values for IZA and DEEM frameworks based on the full SOAP power spectrum with an environment cutoff of 6.0 Å. (c) Receiver operating characteristic (ROC) curve for the IZA *vs.* DEEM SVM classification with 6.0 Å SOAP, as the decision function boundary is swept through decision space as shown by green arrows in (a), (b), and (c). The inset in (c) shows the confusion matrix for the two-class IZA *vs.* DEEM classification using the full SOAP power spectrum, and (d) similarly shows the four-class confusion matrix, with darker shading indicating a greater proportion of the class-wise predictions. The superscripts ^†^ in the confusion matrix labels refer to predicted classifications, and the labels TP, FP, TN, and FN indicate true positive, false positive, true negative, and false negative classifications, where the DEEM and IZA frameworks are denoted as the positive and negative classes, respectively.

Fig. 2 shows how the Zeolite Sorting Hat works and displays its performance in classifying real (IZA, red) and hypothetical (DEEM, blue) frameworks. Fig. 2(a) illustrates how the support vector machine operates by using toy data, in which each dot or square represents the (schematic, 2D) feature vector for a given framework, and where the decision boundary location can be adjusted to optimize the classification (represented by a green arrow). The blue/white/red shading in Fig. 2(a) represents a continuous decision function with negative and positive values corresponding to toy stand-ins for IZA and DEEM, respectively, illustrating the nuanced classification provided by the Zeolite Sorting Hat. Fig. 2(b) reveals the actual histogram of decision function values for the two-class IZA/DEEM sort obtained from the full SOAP power spectrum including two- and three-body correlations within a distance cutoff of 6.0 Å. The histogram is clearly bimodal, indicating that the IZA and DEEM datasets are indeed distinguishable *via* the Zeolite Sorting Hat, a striking contrast to the failure of unsupervised learning shown in Fig. 1. We also see small areas of overlap between IZA and DEEM decision function values; these are important for suggesting IZA-like DEEM frameworks that may be targets for synthesis according to our synthesizability hypothesis. Fig. 2(c) quantifies the performance of the Zeolite Sorting Hat through the receiver operating characteristic (ROC) curve and optimal confusion matrix. The ROC curve optimizes sorting accuracy with respect to the location of the decision boundary (green arrows in Fig. 2) by maximizing the rate of true positives while minimizing false positives. The performance of the sorting procedure can be further assessed by examining the confusion matrix, which consists of rows and columns that represent the classes in which the data is to be sorted (IZA and DEEM in our case). The diagonal terms count the cases in which the prediction and reality match, while the off diagonal terms represent the number of failed predictions. The best Zeolite Sorting Hat performance is shown by the confusion matrix inset in Fig. 2(c), revealing that 102/115 (89%) of the IZA frameworks and 95% of DEEM frameworks are correctly classified. This excellent sorting performance is remarkable given the substantial overlap in energy- and structure-spaces shown in Fig. 1, and substantiates our simplifying assumption of treating the unlabeled DEEM as a dataset containing (mostly) decoys.

The successful two-class (DEEM/IZA) sort prompts the investigation of the even more challenging four-fold (DEEM/IZA1/IZA2/IZA3) classification: a prediction of the reference composition based exclusively on the structure of the pure SiO_2_ framework. Note that all frameworks were treated as all-silica for the purpose of our work, so the success of the four-fold classification cannot be taken for granted. The optimal confusion matrix of the four-way classifier (Fig. 2(d)) demonstrates that the Zeolite Sorting Hat is also successful at this more difficult task. The distinction between IZA1 (all-silica) and IZA3 (no-silicon) is nearly perfect, and most of the incorrect classifications involve IZA2. This is consistent with the fact that our definition for IZA2 encompasses a broad range of compositions from high-silica alumino-silicates (some of which have also been synthesized as pure silica frameworks) to low-silicon silico-aluminophosphates. Thus, the imperfect classification of mixed-composition frameworks can be seen as a verification that the model is not overfitted, and reflects some of the nuances that are not captured by our somewhat artificial clear-cut splitting of the houses. It is also intriguing to note that most of the IZA being misclassified as DEEM belong to the no-silicon IZA3 house, while the all-SiO_2_ IZA1 entries are never mistaken for a hypothetical framework, which is consistent with the fact that IZA3 consists of exotic frameworks that are more diverse and less typical than the all-silica zeolites, as well as farther in their chemistry from the silicate representation that was used for all frameworks. The success of this four-fold sort opens the door to recommending synthesis compositions for IZA-like DEEM structures.

The confusion matrix in Fig. 2(c) shows that ≈15 000 DEEM frameworks are misclassified as IZA. To further narrow down the subset of DEEM structures for which synthesis should be attempted, we augment the notion of similarity generated by the Zeolite Sorting Hat with the concept of thermodynamic stability. The importance of thermodynamic stability (a rough estimate of which is given by the lattice energy^34^) was underscored by a recent machine learning study showing that synthesizable zeolite phases correlate with their thermodynamic stabilities.^35^ However, as discussed above, Fig. 1(a) shows that naively using lattice energies to identify synthesizable DEEM frameworks is insufficient because of the significant overlap between IZA and DEEM energies. Furthermore, most zeolites are only metastable, their synthesis being made possible by carefully chosen thermodynamic conditions.

The convex hull construction is often used in materials discovery as a proxy for the stability of a phase subject to *e.g.* fixed composition, or a prescribed molar volume.^36^ One could imagine building convex hulls in which the parameters that determine the synthesis conditions play the role of generalized thermodynamic constraints, akin to composition and volume. Their diversity, however, makes it all but impossible to map them on a single, clearly-defined stabilizing order parameter. For this reason, we have used a generalized convex hull (GCH) construction, which uses data-driven coordinates that are computed as a function of the atomic configurations, and provide a low-dimensional description of the structural diversity of the frameworks. The idea is that two structures that are very different from each other respond in different ways to the application of a thermodynamic constraint, so that by designing appropriate synthetic conditions it might be possible to stabilize, and therefore make, the structure which is less stable in terms of lattice energy. Frameworks with very similar structure, instead, respond similarly to any thermodynamic forcing, and so among neighboring configurations only those with low lattice energy can be synthesized. The GCH construction provides a quantitative way to apply this concept, as discussed in ref. 37, by defining the stability as the vertical distance from the hull built from the data-driven coordinates (the “hull energy”).

The GCH does not, however, say what synthesis parameters correlate with the data-driven coordinates, nor how to optimize the data-driven coordinates so that they correlate with known synthesis parameters. To determine a coordinate space for the GCH that can be loosely interpreted in relation to known synthesis conditions, we use the decision functions of the 4-way Zeolite Sorting Hat, and apply the method of principal covariates regression (PCovR),^38,39^ which optimizes a lower-dimensional space that supports classification by the Zeolite Sorting Hat, and simultaneously resolve as well as possible the structural differences between frameworks. We produce a 2D latent space for the GCH which separates as well as possible DEEM and each of the three IZA houses, and take the energetics for the GCH from the same classical forcefield^40^ used by Deem and coworkers. After determining which frameworks define the vertices of the GCH, we can compute the “hull energies”, and use them as an energetic measure of synthesizability in the sense discussed above.

**Fig. 3:**
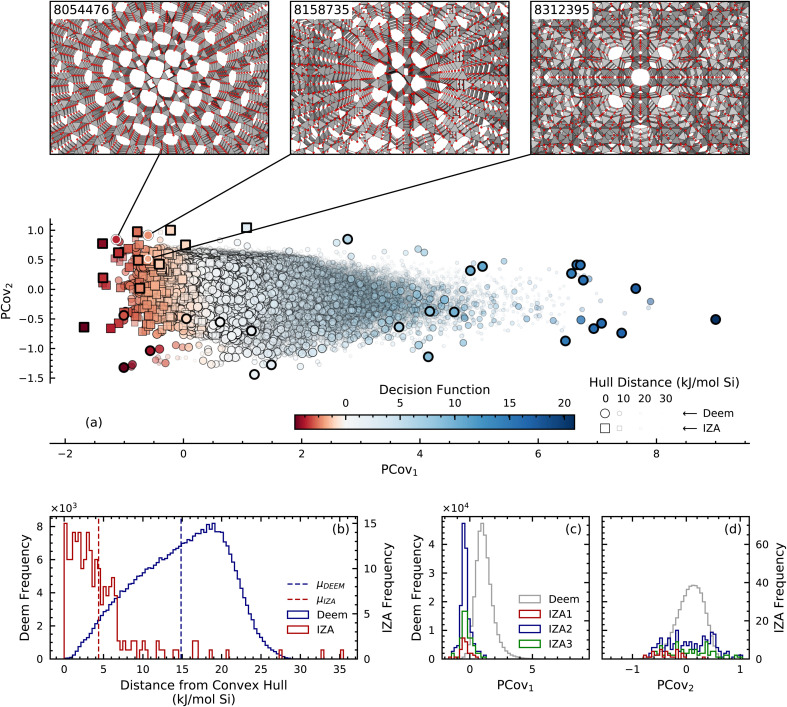
(a) First two components of the PCovR projection based on the four-class decision functions with IZA (square) and DEEM (circle) frameworks colored according to two-class decision function value (red: IZA-like; blue: DEEM-like). Larger, more opaque points lie close in energy to the GCH; hull vertices are indicated *via* points with thick black borders. (b) Histogram of the energy distance to the convex hull for the IZA and DEEM frameworks. (c) and (d) Histograms of the PCovR component values for the IZA houses and DEEM.

A visualization of the resulting GCH construction is given in Fig. 3(a), in which each IZA and DEEM framework is respectively plotted as a single square or circle and is colored according to its two-class DEEM/IZA decision function value. The points are also sized and given an opacity corresponding their hull energies: larger, more opaque points are those closer to the GCH. The hull vertices are indicated *via* thick black borders, and are energetically stable relative to the frameworks that are close to them in PCovR space. This local stability implies that—subject to appropriate thermodynamic constraints—it might be possible to stabilize all the hull frameworks, including those which lie to the right of the plot. However, the conditions needed to stabilize frameworks that are very distinct from any IZA structure are likely to be completely different from those used in the synthesis of any currently known zeolite. Thermodynamic stability and structural similarity to known zeolites should be used simultaneously to assess the synthesizability of a candidate structure. The DEEM frameworks that show the most promise according to our criteria, then, are those that are misclassified as IZA and lie close to the GCH, *i.e.*, the large, opaque, red circles in Fig. 3(a).

The utility of the GCH construction based on a PCovR latent space is evident upon comparing Fig. 3(b)–(d) with Fig. 1(a)–(c). While the difference in mean IZA and DEEM lattice energies in Fig. 1(a) is only 5 kJ mol^−1^ Si, the mean hull energies shown in Fig. 3(b) differ by more than 10 kJ mol^−1^ Si, with most IZA frameworks either on or very close to the GCH, thus confirming that the energy distance from the convex hull is a more selective filter for thermodynamic stability than the bare lattice energy. Fig. 3(c)–(d) also reveal the advantage of the PCovR space over the PCA space (Fig. 1(b)–(c)) in arranging the IZA and DEEM frameworks: the first PCovR component correlates strongly with DEEM/IZA decision-function values, and therefore highlights the structural distinction between the real and hypothetical frameworks—an arrangement that is largely absent from the PCA. Furthermore, the second PCovR component (Fig. 3(d)) roughly organizes the IZA frameworks according to their compositional (house) classification: there is minimal overlap between the all-silica (IZA1) and no-silicon (IZA3) frameworks, and the frameworks that contain both Si and other tetrahedral (“T”) atoms (IZA2) overlap with both the all-silica and no-silicon frameworks. The separation into IZA houses through the Zeolite Sorting Hat shown by the confusion matrix (Fig. 2(d)) is actually better than what can be visually inferred from the histogram in Fig. 3(d) because the classification is applied in the full-dimensional SOAP space and not in that obtained by 2D PCovR, which trades-off some information to achieve a low-dimensional representation. This visualization also demonstrates that our analysis based on the value of the decision function allows us to recover some of the nuance that is inherent to the zeolite conundrum. The misclassified IZA structures lay close to the decision boundary, suggesting the possibility of interpreting the two-class decision function as an indication of how “typical” a putative zeolite framework is: indeed, we recall that most of these misclassified structures belong to the IZA3, no-silicon house, and that they can be seen as “borderline” cases with unusual geometries. Similarly, the greater spread in the distribution of the IZA2 structures is consistent with the broad definition of this class.

The GCH construction leaves us with approximately 4600 DEEM structures that are classified by the Zeolite Sorting Hat as belonging to IZA and that lie within a 5 kJ mol^−1^ window from the hull. From energetic and structural perspectives, these frameworks appear as likely to be synthesizable as structures that have been made already. While selecting among these worthwhile candidates for synthesis can be achieved by ranking the structures based on their GCH distances, a more application-oriented selection can be performed by introducing a secondary filtering criterion. To demonstrate this approach, we stratify the DEEM dataset in terms of molar volume and select the IZA-like DEEM candidate closest in energy to the hull within each of the 55–60, 60–65, and 65–70 Å^3^/Si ranges, which cover the upper end of the distribution of molar volumes for known IZA structures. This stratified search can be intuitively visualized using the chemiscope structure–property explorer;^41^ a link to the visualizer is included in ESI† for readers to explore.

The structures of the resulting three promising DEEM frameworks are highlighted in the insets of Fig. 3(a). Two (8158735 and 8054476) are classified by the Zeolite Sorting Hat as belonging to IZA2, *i.e.* as aluminosilicates, and the third (8312395) is classified as IZA3, a zeolite containing no silicon, suggesting synthesis as an aluminophosphate. Framework 8158735, the candidate within the 55–60 Å^3^/Si range, is closest to the IZA framework THO in SOAP space and exhibits rings of 3, 4 and 8 T atoms; THO is similarly composed of 4- and 8-rings. The candidate within the 60–65 Å^3^/Si range is the triclinic framework 8054476, having SBN as its closest IZA neighbor. Like SBN, framework 8054476 contains 4-, 8- and 9-member rings. The DEEM framework in the largest volume category that is closest in energy to the GCH is structure 8312395, which shares many structural similarities to its nearest IZA neighbor RHO: both frameworks contain 4-, 6-, and 8-member rings. Additional discussion of the similarities of these three candidate DEEM frameworks and their nearest IZA analogues is given in the ESI.†

Let us outline how we foresee our analysis can provide some indications to facilitate the design of a synthetic route, taking DEEM zeolite 8158735 (DEEM-A) as an example. For this framework, the Sorting Hat points to synthesis as an aluminosilicate. We note that the closest IZA cousin to DEEM-A is THO, which can be fabricated using a hydrothermal treatment of precursor gels made from Na_2_O, CaO, Al_2_O_3_, and SiO_2_.^42^ Obviously using this exact treatment will yield THO and not DEEM-A, but it serves as a data-driven initial condition for an optimization of the reaction mixture and any structure directing agents, perhaps through the use of docking calculations.^43^ Armed with the result of such an optimization for DEEM-A, we would have an excellent starting point in the synthesis conditions space to find a successful protocol for its synthesis in a few synthesis conditions iterations; the same logic and considerations apply for hypothetical zeolites 8054476 (DEEM-B) and 8312395 (DEEM-C). The above recommendations show how our data science results fit into the ecosystem of zeolite synthesis design.

**Fig. 4:**
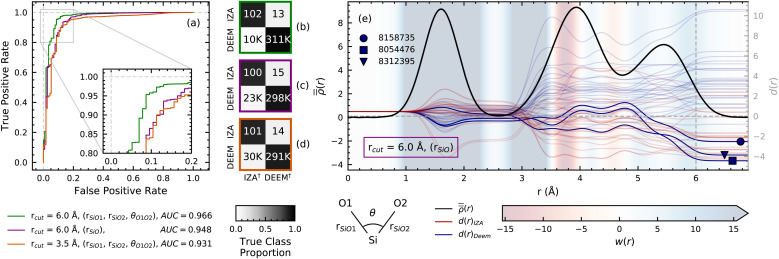
(a) ROC curves of SVM classifications for three “knock-out” models based on a limited set of structural correlations between a Si atom and neighboring O atoms. The models include angular and radial correlations up to 6.0 Å (green), only radial information (purple) and radial and angular correlations limited to 3.5 Å (orange). The corresponding confusion matrices are shown in panels (b)–(d). (e) Class-averaged IZA and DEEM real-space densities based on Si–O correlations (black line) plotted together with the decision traces *d*(*r*) for 25 DEEM frameworks (faded blue lines) and 25 IZA frameworks (faded red lines). The *d*(*r*) for the three highlighted frameworks from Fig. 3 are also plotted as fully opaque lines and are labeled using symbols. The line *d*(*r*) = 0 indicates the decision boundary: the top half corresponds to DEEM predictions, the bottom half to IZA. The background coloring representing the SVM weights *w*(*r*) is subject to a threshold to better show sign changes: weights falling outside the colorbar limits are assigned the color at the corresponding end of the colorbar.

The Zeolite Sorting Hat's ability to discriminate between IZA and DEEM, and among different reference compositions of IZA, represents a breakthrough that begs a fundamental question: what aspects of zeolite structure are critical to these discriminating powers? Additionally, how do the features that emerge from an analysis of the SOAP-based models compare with some of the structural discriminants that have been postulated as inputs for previous real and hypothetical zeolite comparisons? To reveal key structural discriminants, we first performed an ablation study in which we built several “knock-out models” that use only a subset of the structural features. We repeat the two-class DEEM/IZA sort to determine the impact on classification performance of (i) restricting the range of correlations to first neighbors (up to 3.5 Å), (ii) considering only radial information on pair correlations, and (iii) using only some of the three-body angular correlations between Si and O neighbors. We show three representative models in Fig. 4(a)–(d), and report a more systematic investigation in the ESI.†

Restricting the range of correlations or discarding all angular information leads to a degradation of classification performance, indicating that the structural features that distinguish real and hypothetical zeolites involve angular correlations and patterns in the relative positions of second and third neighbor atoms, *i.e.*, at length scales beyond the typical indicators that have been hypothesized in previous studies,^15–22^ and in line with the T site-fifth neighbour distance tested by Perez and coworkers.^20^ It is also interesting to see that a model that uses only 6.0 Å 3-body Si–O correlations, rather than also Si–Si correlations, leads to a noticeable improvement in resolving power: the number of misclassified DEEM structures drops by 30%. This underscores the data-limited nature of this exercise, that requires finding a set of descriptors that is sufficiently informative, yet not overcomplete.

Second, our use of linear constructs—SOAP vectors and linear support vector machines—allows us to recast the Sorting Hat in a “real space” form to elucidate the spatial weights that distinguish real and hypothetical zeolites. The decision functions are then obtained by summing the values of these weight functions over all pairs and triplets of atoms in a structure. For a purely radial model, the decision process can then be interpreted as the incremental construction of a “decision trace” *d*(*r*) (see Methods) that, for *r* → ∞, gives the decision function value used for classification. The length scales at which *d*(*r*) undergoes large changes are those that dominate the classification.

Fig. 4(e) shows that, for most DEEM frameworks, *d*(*r*) settles to plateau positive values around *r* = 3.5 Å, corresponding to the onset of second-neighbor Si–O correlations (3–4 Å). Known IZA frameworks show an opposite behavior, drifting towards negative decision values in the same region. The role played by these second-neighbor Si–O correlations manifests itself in the sharp change of *w*(*r*) from positive to negative values at around 3.5 Å, indicating that frameworks in which the second-neighbor Si–O peak appears at shorter-than-average distances favor a DEEM prediction, and *vice versa* for IZA. We thus conclude that second-neighbor Si–O distances are the most clearly discernible structural feature that differentiate IZA and DEEM frameworks. More subtle structural correlations involving angular information and third-neighbor distances are needed to achieve classification accuracies above 90%, making an automatic data analysis preferable to *ad hoc* heuristics.

**Fig. 5:**
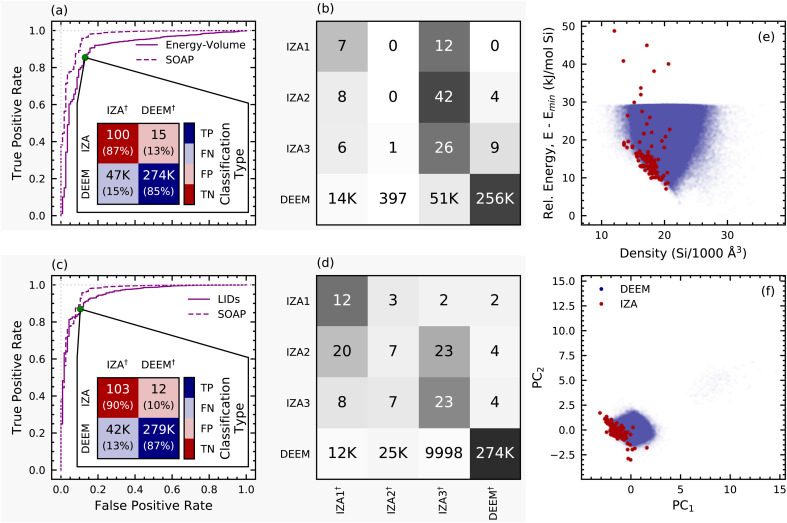
ROC curves and confusion matrices for the energy–volume (a) and (b) and LID (c) and (d) descriptors, analogous to Fig. 2(c) and (d). The ROC curve for the SOAP descriptor in Fig. 2(c) is plotted alongside the ROC curves for the energy–volume and LID descriptors for comparison. (e) Energy–density plot for the IZA and DEEM frameworks, illustrating the discriminating power of using framework energy and volume as input to a support vector classifier. (f) Two-component PCA decomposition of the nine LID features, also visually demonstrating their potential to distinguish IZA frameworks from DEEM.

To contextualize the discriminating power of an SVM based on SOAP features, we also constructed SVM models based on (i) the molar volumes and energies of the zeolite frameworks, and (ii) the local interatomic distances (LIDs) of Li *et al.*;^17^ the ROC curves and confusion matrices of these two “classical” models are shown in Fig. 5. Despite their simplicity, the classical models perform well in separating IZA from DEEM, with AUC values of 0.91 and 0.95, respectively, in comparison with 0.97 obtained from the full SOAP approach (Fig. 2). Another important way to compare these descriptors is *via* the number of misclassified DEEM frameworks, which we want to make as low as possible to focus on the most feasible hypothetical structures. The full SOAP model (6.0 Å power spectrum) misclassifies 15 000 DEEM frameworks, that can be reduced further to 10 000 by dropping redundant descriptors, as shown in Fig. 4(a). The volume/energy and LID descriptors misclassify 47 000 (Fig. 5(a)) and 42 000 DEEM frameworks (Fig. 5(c)), respectively, thus diluting the search for feasible DEEM frameworks with low-value targets because of the incompleteness of these classical structural descriptors. Also notable is the fact that the energy–volume and LID models perform more poorly in the four-class exercise compared to the top SOAP models (compare Fig. 5(b) and (d) to Fig. 2(d)).

In comparison with the SOAP-based models (see ESI†), the energy–volume descriptor is outperformed by all but the models containing only radial information on the Si atoms. In contrast, the LID-based SVM outperforms all but the best 3.5 Å SOAP models, but is outperformed by all of the 6.0 Å power spectrum models, which contain 3-body correlations that are missing in LIDs. Overall, these results suggest that traditional representations of zeolite frameworks such as energy, volume, and local interatomic distances can be used to effectively distinguish IZA and DEEM frameworks; however, the longer-range and angular correlations afforded by an appropriately constructed SOAP representation provide additional improvements that reduce the number of misclassified DEEM structures by more than a factor of 3 or 4, considerably refining the space of candidate structures.

## 3 Conclusions

Synthesizing new zeolites is both an exciting intellectual challenge, and a technological quest with high potential rewards. The huge databases of computationally-proposed zeolites stand in striking contrast to the 255 known framework topologies (IZA), configuring a highly asymmetric problem that hinders the solution of this zeolite conundrum by brute-force applications of data science. We developed and applied a multi-pronged strategy for sifting through the hypothetical zeolites in search of the most promising candidates for synthesis. This “Zeolite Sorting Hat” tackles data scarcity by using flexible and unbiased SOAP structural descriptors as inputs, and relatively simple and robust linear classification algorithms *via* support vector machines to reach a 95% accuracy in distinguishing real and hypothetical structures. The 5% of hypothetical structures that are recognized as “real” by the Zeolite Sorting Hat then become promising candidates for synthesis. A thermodynamic stability criterion provides an additional filter, and together with stratification by framework density leads us to propose three leading hypothetical candidates for synthesis. By further partitioning IZA frameworks into “zeolite houses” based on known reference compositions, and by quantifying geometric proximity to existing materials, we provide guidance for synthetic efforts at fabricating new zeolites. The principled choices we made in the architecture of the Zeolite Sorting Hat also affords a degree of interpretability in the classification process, pointing to the importance of second-neighbor Si–O distances as the leading factor that distinguishes real and hypothetical frameworks.

As it is the cases for many synthetic tasks, making zeolites is a form of art, guided by experience, chemical intuition and serendipity. The Zeolite Sorting Hat introduces data-driven techniques and rational design^44,45^ into the process of selecting candidates that we hope will accelerate the rate of discovery, which in turn will improve the predictive capabilities of the model in a positive feedback mechanism that will progressively take the guesswork out of zeolite synthesis.

## 4 Methods

Real zeolite structures were obtained as CIF files from the online IZA database (https://www.iza-structure.org/databases/); of the approximately 250 structures in the database, we consider only those that are fully connected (as of early 2021), totalling 230 structures. For the hypothetical zeolites we use the dataset constructed by Pophale *et al.*^7^ All the IZA structures were optimized following the same procedure previously considered for the DEEM zeolites. The structural relaxation was performed with GULP^46^ using a modified version of the Sanders–Leslie–Catlow (SLC) potential^40^ to overcome the negative energy divergence due to the Buckingham contributions for *r* → 0 (see ESI†). The same forcefield was also used to evaluate the framework energies for the IZA structures in their optimal configurations. Since the DEEM frameworks are already relaxed with the SLC forcefield, we computed their energies though a relaxation of the atom shells only, keeping the cell and cores fixed. Our computed energies were in good agreement with those obtained by Deem and coworkers, except for five frameworks which were thus discarded from all of our analyses. We have also discarded DEEM frameworks that we found to be identical to an IZA framework. To establish this, we evaluated the Euclidean distance between the full power spectrum SOAP feature vectors with an environment cutoff of 6.0 Å (more details can be found in the ESI†).

Local interatomic distances (LIDs), as defined by Li and coworkers,^17^ were computed on the Deem structures and the optimized IZA frameworks using the pymatgen package.^47^ With this approach, each framework is represented by a nine-component vector: for each kind of distance (T–O, T–O–T, and O–T–O), we include the mean value, the standard deviation, and the range. The values are computed from the list of all the distances of the same kind found in each framework.

SOAP representations were computed using librascal^48^ for two different environment cutoffs: 3.5 Å and 6.0 Å for each Si-centered environment (that is, for every Si atom in a given framework). For our structure-based analyses, we define the SOAP representation of a given framework as the average over the SOAP vectors corresponding to each of its Si atoms. Further details of the SOAP calculations can be found in the ESI.†

Molar energies, volumes and compositions were predicted by optimizing the mean absolute error of the target property predictions *via* ridge regression.

Two-class (DEEM/IZA) and four-class (DEEM/IZA1/IZA2/IZA3) linear kernel SVM models were built using scikit-learn^49^ to distinguish the IZA frameworks from the DEEM. The SVM models were constructed for each combination of SOAP environment cutoff (3.5 Å or 6.0 Å), *n*-body correlation (two-body radial spectrum and three-body power spectrum), and atom–atom correlations (Si–Si correlations, Si–O correlations, and Si–Si and Si–O correlations for the radial spectrum; and Si–Si–Si correlations, Si–O–Si correlations, and Si–O–O correlations and all combinations thereof for the power spectrum). The real-space expansion of the SOAP vectors was performed through a sum over the product of “contracted”^50^ Legendre DVR radial basis functions and spherical harmonics based on Legendre polynomials (for the power spectrum) with the SOAP vectors summed over the expansion orders and angular index. The optimization target of the SVM was the class-balanced accuracy. To lend support to the validity of the class distinctions learned by the SVM, we assigned a set of random labels to the DEEM frameworks and subsequently attempted to classify them based on the randomly assigned labels. The SVM was unable to learn the random labels, suggesting that the learned distinctions between the IZA and DEEM frameworks are indeed due to genuine differences in their structural characteristics. The “decision trace” *d*(*r*) is defined by combining the smoothed radial correlation function *ρ*(*r*) (the real-space counterpart of the pair descriptor associated with each structure) and a weight *w*(*r*) that is also a function of distance, yielding1
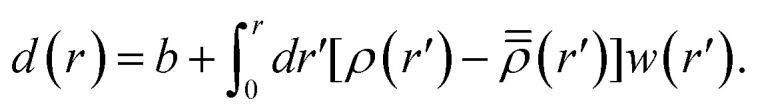
*d*(*r*) is defined to also include the SVM intercept *b* and the class-averaged radial correlation function 
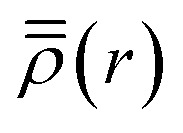
, so that the decision trace provides the value of the decision function based only on contributions between 0 and *r*; lim_*r*→∞_*d*(*r*) gives the value which is ultimately used for classification.

The PCovR models were constructed using the same feature data and decision values as the corresponding SVM models. The optimization target was the PCovR loss (the sum of regression and projection losses) based on a three-component latent space. The convex hull was constructed in the space defined by the framework energies and the first two PCovR components for the model based on the full 6.0 Å SOAP power spectrum feature vectors and the corresponding four-class decision functions for the classification exercise on these same feature vectors.

The train-test splits of the datasets were slightly modified based on the ultimate goal of the machine learning exercise (see details in the ESI†). To account for the imbalance in the class populations, we employed class-specific misclassification penalties in the SVM models: the penalty for a given class is weighted inversely to the true class proportion in the train set. In contrast, class imbalance in the PCovR models was accounted for through replication of minority samples to achieve approximate class parity. This approach was preferred over undersampling the majority class, because the smallest number of minority class samples in a given training fold was very low, *i.e.* fewer than 20 structures.

## Data availability

The data used as input to the machine learning workflow, including the (optimized) atomic structures of the IZA and DEEM frameworks, house classifications, framework energies and volumes, and LID descriptors, as well as a chemiscope visualization^41^ of the Generalized Convex Hull used to identify promising synthesis candidates are available on the MaterialsCloud Archive at: https://doi.org/10.24435/materialscloud:sd-j6.

## Author contributions

R. S., G. P., and B. A. H. curated the structural databases and performed preliminary analyses; G. P. and B. A. H. carried out force field calculations for the zeolite frameworks; B. A. H. constructed the machine learning models and assembled figures; all authors contributed to the conceptualization of the study and the writing of the manuscript.

## Conflicts of interest

The authors declare no competing interests.

## Supplementary Material

DD-001-D2DD00056C-s001
