# Supplementary material for: Ranking the synthesizability of hypothetical zeolites with the sorting hat
Source: Digit Discov. 2022 Oct 12;1(6):779–89. doi: 10.1039/d2dd00056c (PMC9721151; doi:10.1039/d2dd00056c)
Supplement: DD-001-D2DD00056C-s001 [file DD-001-D2DD00056C-s001.pdf]

Electronic Supplementary Material (ESI) for Digital Discovery.
This journal is © The Royal Society of Chemistry 2022

Supporting Information: Ranking the Synthesizability of Hypothetical Zeolites with the Sorting Hat: Supplementary Information

Benjamin A. Helfrecht,[†] Giovanni Pireddu,[‡] Rocio Semino,[*,¶] Scott M. Auerbach,[*,§] and Michele Ceriotti[*,†]

†*Laboratory of Computational Science and Modeling, Institut des Matériaux, École Polytechnique Fédérale de Lausanne, 1015 Lausanne, Switzerland*

‡*PASTEUR, Département de Chimie, École Normale Supérieure, PSL University, Sorbonne Université, CNRS, 24 rue Lhomond, 75005 Paris, France*

¶*ICGM, Univ. Montpellier, CNRS, ENSCM, Montpellier, France*

§*Department of Chemistry and Department of Chemical Engineering, University of Massachusetts Amherst, Amherst, MA 01003 USA*

E-mail: rocio.semino@umontpellier.fr; auerbach@umass.edu; michele.ceriotti@epfl.ch

Data preparation

The IZA zeolite structures were optimized via a modified version of the Sanders-Leslie-Catlow (SLC) potential.[S1] We introduced a linear extrapolation, starting at the first inflection point and yielding finite potential values for $r = 0$ to overcome the negative energy divergence due to the Buckingham contributions for $r \to 0$. The slopes chosen for the extrapolations were

calculated from the derivative of the Buckingham contributions at the inflection points. To compute the energies, we performed a constant pressure optimization of the cell, atom cores, and atom shells. In the cases where the constant pressure optimization did not converge, we performed a constant volume optimization from scratch, for which only the geometry of the atom cores and shells were optimized.

For the hypothetical frameworks in the DEEM database, we generally found very good agreement between our energies (obtained by optimizing only the atom shells) and those calculated by Pophale et al.[S2], with a mean absolute error of 0.08 kJ/mol Si, a median absolute error of 0.07 kJ/mol Si, and a standard deviation in the discrepancy of 0.9 kJ/mol Si.

We computed the SOAP vector for each atomic environment centered on a Si atom, considering two cutoffs: 3.5 Å and 6.0 Å. For each environment cutoff, we computed both the two-body radial spectrum and the three-body power spectrum with a Gaussian atom width of 0.3 and a cutoff transition width of 0.3. For both the power spectrum and radial spectrum we used a basis of 8 radial functions, truncated through a PCA of the density coefficients[S3] from a radial basis of 32 Legendre polynomials in the discrete variable representation (DVR). The power spectrum representations additionally use 9 angular functions in the density expansion.

Data Cleaning

Fig. S1 shows a histogram of the discrepancy between our calculated GULP energies and those reported for the frameworks in the DEEM database. The five frameworks for which the discrepancy was greater than 10 kJ/mol Si are highlighted with their database ID number, and were subsequently discarded from all of our analyses.

Fig. S2 presents a histogram of the distances between the full 6.0 Å power spectrum SOAP vectors of every DEEM framework with every IZA framework, where the SOAP feature vector for a structure is an average over the SOAP vectors for all of the Si-centered envi-

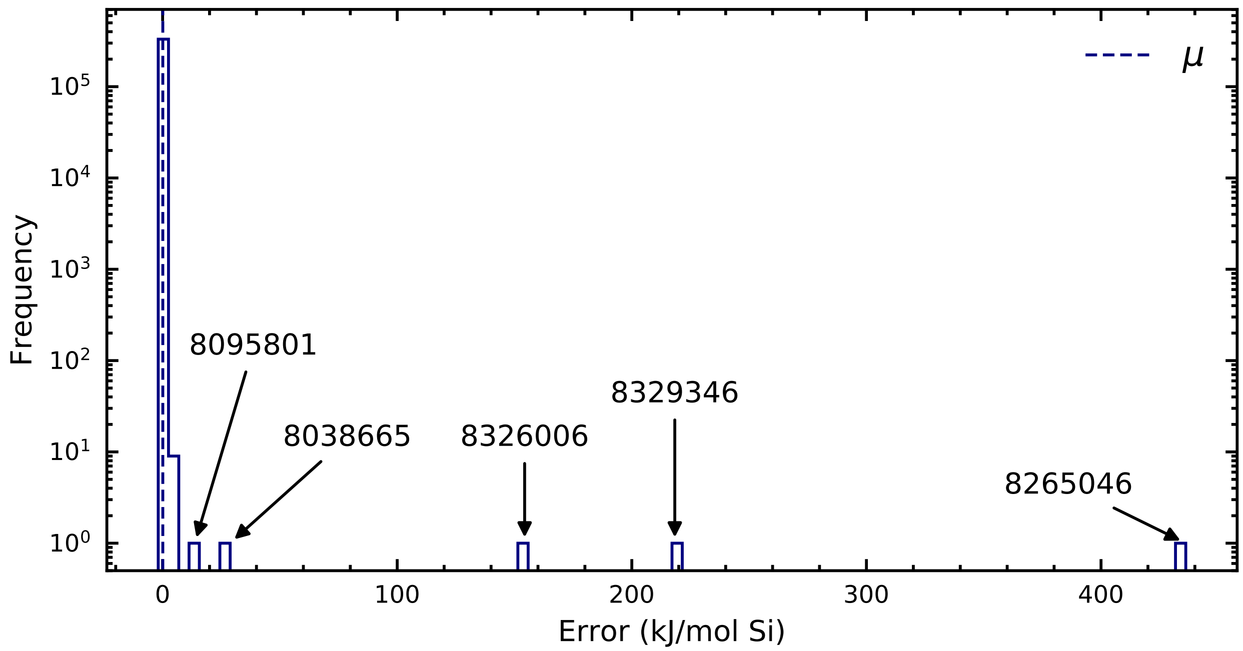

Figure S1: Histogram of errors representing the discrepancy between our GULP calculations of the framework molar energy for the approximately 330k structures in the DEEM database of hypothetical zeolites. Structures with energy discrepancies larger than 10 kJ/mol Si are highlighted in the histogram and were discarded from the machine learning analyses.

ronments within the structure. The purpose of the histogram is to identify those frameworks within the DEEM database that are "identical" to an IZA framework, which is suggested by very small DEEM-IZA distances. The histogram in Fig. S2 shows two peaks, one between 10^{-7} and 10^{-6} and the other at 10^{-3}. As a result of this observation, we set the distance cutoff for identifying "identical" frameworks in the "no-man's land" between the two peaks. Following this reasoning, 5×10^{-6} appears to be a reasonable choice.

Train/Test Split

The train/test split used in the different parts of the machine learning workflow vary slightly based on the ultimate goal of the particular exercise.

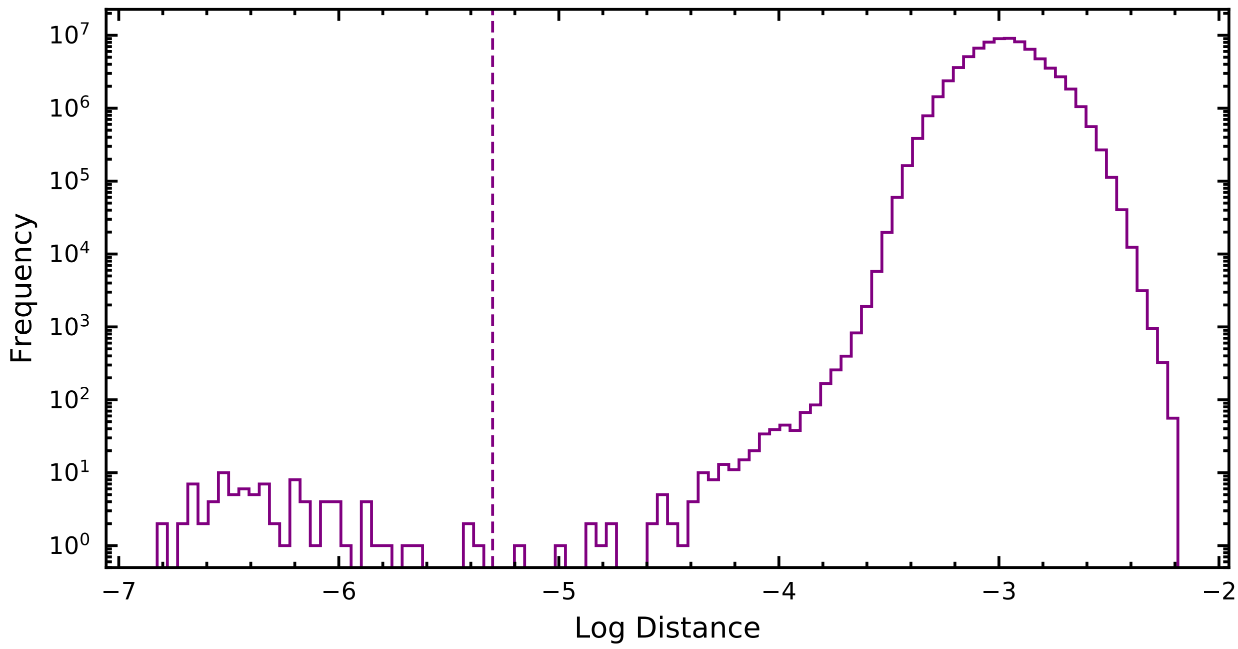

Figure S2: Histogram of distances between the 330k frameworks in the DEEM database of hypothetical zeolites and the 230 IZA structures. The distance is taken to be the Euclidean distance between the average SOAP vectors (using a 6.0 Å cutoff) between the frameworks. The distance cutoff for declaring structures as “identical” is 5×10^{-6}.

Linear regression of molar volumes and energies, PCA decomposition

The training set includes all 10,000 frameworks used in Helfrecht et al.[S4], and the linear regression test set includes all 230 IZA frameworks and 250 DEEM frameworks selected randomly from the full 330k dataset and that are not present in the train set, excluding the five frameworks with large energy discrepancies. None of the frameworks in the training set of 10,000 are subject to elimination based on their energy discrepancy.

Support vector machine, PCovR

The training set includes the DEEM frameworks used in Helfrecht et al.[S4] that are not also determined to be identical to an IZA framework (through the Euclidean distance between SOAP vectors, see above) and that have an energy discrepancy less than 10 kJ/mol Si, and

half of the IZA frameworks after excluding RWY. The DEEM frameworks that are identical to IZA are eliminated to avoid conflicting class labels to the SVM for nearly the same feature vector. As a result of these criteria, the training set contains 9,999 DEEM frameworks and 114 IZA frameworks for a total of 10,113 structures. The test set contains the remaining 321,082 DEEM frameworks and the remaining 115 IZA frameworks, for a total of 321,197 structures.

Convex hull construction

The convex hull construction is built using all of the IZA and DEEM frameworks not eliminated during the data cleaning step, i.e., the union of the train and test sets used for the SVM and PCovR models. The construction is based on that of the Generalized Convex Hull,[S5] though it is deterministic in nature, rather than probabilistic. In other words, we construct the convex hull in the space defined by the framework energies and the first two PCovR components for the PCovR model based on the full 6.0 Å SOAP power spectrum feature vectors and the corresponding four-class decision functions for the classification exercise on these same feature vectors. In contrast to the original GCH framework, however, no repeated sampling or pruning of the hull vertices is performed.

Linear regression of compositions

For our linear regression exercise on the structure compositions (fraction of Si), we use as the training set the 114 IZA training structures used in the SVM and PCovR models. This model is used to predict compositions for all of the DEEM frameworks, and we evaluate the performance of this model on the 115 IZA frameworks in the test set of the SVM and PCovR models.

IZA Predictions from DEEM

The overlap between the IZA and DEEM datasets can be assessed with both supervised and unsupervised learning. In Fig. S3, we show a projection of all IZA and DEEM frameworks (minus the DEEM frameworks with large energy discrepancies) onto the first three principal components of the 10,000 DEEM frameworks from Ref.[S4], with a full 3D projection in the upper panel and individual projections onto the xy, yz, and xz planes in the bottom panels. The IZA frameworks, colored by class designation, appear clustered toward one end of the PCA space, though still largely within the space occupied by the DEEM frameworks. This overlap suggests that the structural diversity of the DEEM database largely encompasses that of the IZA database. However, the PCA projection only provides a qualitative means of assessing the "IZA-ness", or proximity of a DEEM framework to the IZA space. In contrast, a support vector machine does provide this notion of proximity quantitatively through the decision function.

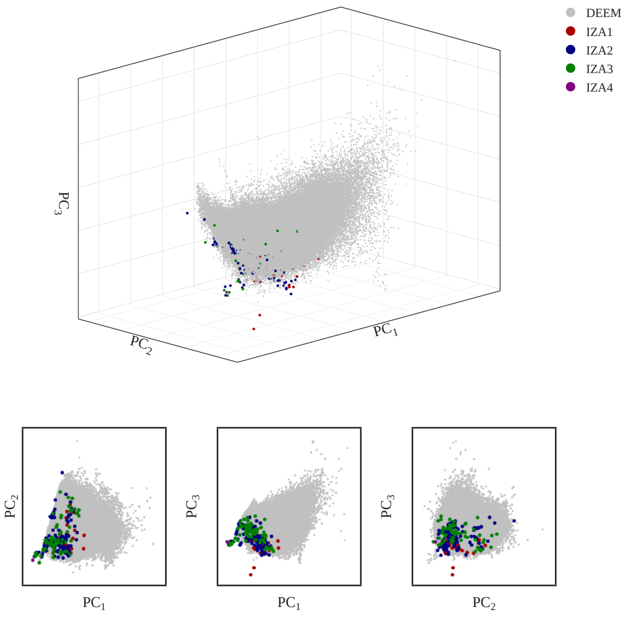

Figure S3: PCA projection of IZA and DEEM structures onto the first three principal components defined by a subset of 10,000 DEEM frameworks.

It is also possible to obtain a baseline understanding of the overlap between the IZA and

DEEM spaces through a regression exercise. Table S1 shows the mean absolute errors (MAE) of molar volume and molar energy predictions on the IZA frameworks and a comparably sized, randomly selected set of DEEM frameworks from a ridge regression model again trained on the 10,000 DEEM frameworks from Ref.[S4]. Both 3.5 Å and 6.0 Å SOAP power spectrum feature vectors are used to learn the molar volumes and energies. The ridge regression models are able to predict the molar energies of the IZA frameworks across all three subcategories (with the exception of IZA4, i.e., RWY) despite being trained only on DEEM frameworks. Relatively speaking, the models have more difficulty in predicting the molar volumes of the IZA frameworks compared to the energies, though the predictions themselves are not entirely unreasonable, particularly in the 6.0 Å case, again suggesting that there is substantial overlap between the IZA and DEEM SOAP feature spaces.

Table S1: Mean absolute errors for predictions of molar volume (V, units Å^3/Si) and molar energy (E, units kJ/mol Si) from a linear ridge regression model trained on a subset of 10,000 structures from the DEEM database and tested on an unseen set of DEEM and IZA structures. While IZA prediction errors are 1.5–3× larger than for the DEEM structures, the volume and energy predictions are not unreasonable, particularly for the all-silica structures and the models based on a 6.0 Å SOAP representation.

		3.5 Å		6.0 Å	
	n_{test}	V	E	V	E
DEEM	250	2.81	0.65	1.10	0.19
IZA	230	5.30	0.92	1.70	0.18
IZA1	36	4.54	0.98	0.96	0.14
IZA2	125	5.17	0.88	1.57	0.15
IZA3	68	5.38	0.94	1.91	0.23
IZA4	1	44.28	2.02	30.52	1.97

Linear Regression setup

Energies and volumes

Predictor and target data were centered by the (columnwise) mean of the train set and scaled (divided) by the L2 (Frobenius) norm of the centered data divided by the square root of the number of samples. The regularization parameter of the regression is optimized by an order-of-magnitude grid search using five-fold cross-validation with a search range of 10^{-10} to 10^{0}. The optimization targets are the mean absolute errors of the energy/molar volume predictions respectively.

Compositions

The data pre-processing was performed in the same manner as in the regression exercise for the molar volumes and energies, but the regularization parameter was optimized with a search range of 10^{-10} to 10^{5} and two-fold cross-validation. The optimization target is the mean absolute error of the composition predictions.

Principal Component Analysis Setup

For the SOAP-based descriptors, the data were pre-processed in the same way as in the linear regression exercise for molar volumes and energies. The first three principal components were computed. For the "classical" descriptors (energy–volume and LIDs), the individual columns were standardized (i.e., to have mean zero and unit standard deviation) relative to the training set. The first two principal components were computed.

Support Vector Machine Models

Setup

The construction of SVM models accounting for specific correlations or combinations of correlations amounts to using subsets of the SOAP feature vectors (computed with librascal,[S6] which includes a utility for identifying which features correspond to which correlations) for the model training and evaluation. For the four-class case, we constructed one-vs-rest SVM models.

The SOAP feature data are additionally columnwise centered relative to the train set with a weighted mean (using the same weights as for the balanced, class-specific SVM penalties) before being scaled (divided) by the L2 (Frobenius) norm of the centered data divided by the square root of the number of samples in the train set. For the classical descriptors, the individual columns were standardized relative to the training set. We used SVMs from `scikit-learn`[S7] with a convergence tolerance of 10^{-3}. The regularization parameter C (scale factor for the misclassification cost) was optimized with an order-of-magnitude grid search using a stratified two-fold cross-validation (to ensure each fold contained roughly the same proportion of frameworks from each class) with a search range of 10^{-4} to 10^{4}. In the multi-class case, all the individual binary classifiers composing the model share the same hyperparameters; they were not optimized individually. The same folds are used in the cross-validation procedure for each model in the ensemble.

Decision Function Histograms

Fig. S4 is similar to Fig. 2 in the main text, but based on SOAP power spectrum feature vectors with a 3.5 Å environment cutoff instead. The results are quite similar to the 6.0 Å model provided in the main text, though the 3.5 Å model misclassifies a few thousand more DEEM frameworks as IZA.

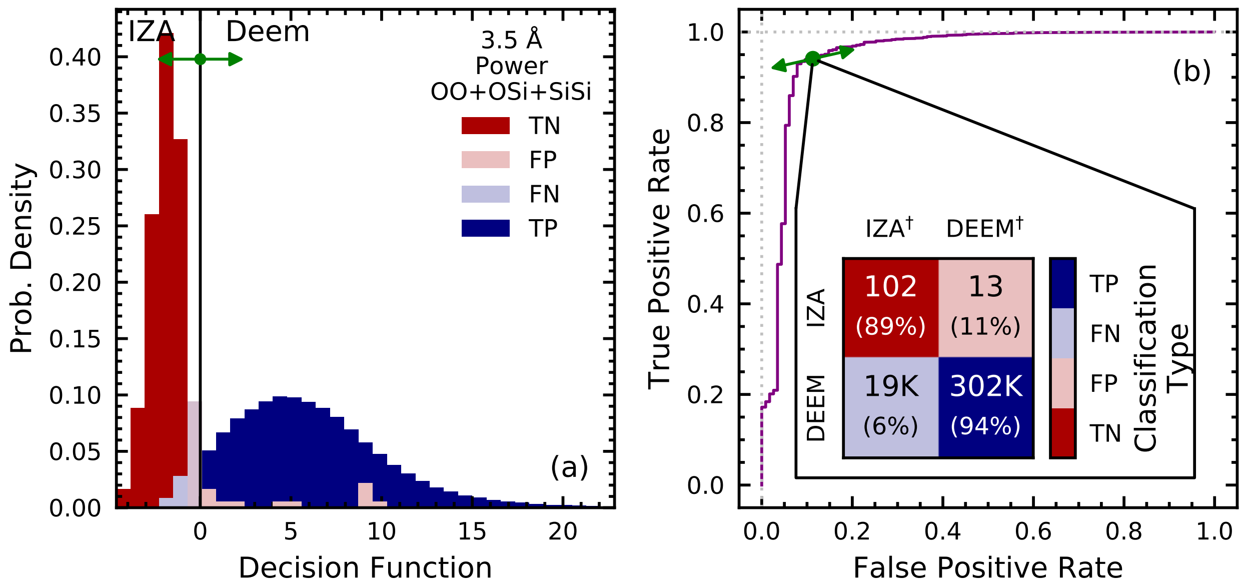

Figure S4: (a) Histogram of decision function values for IZA and DEEM frameworks based on the full SOAP power spectrum with an environment cutoff of 3.5 Å; (b) ROC curve for the IZA vs. DEEM SVM classification based on the same 3.5 Å SOAP representation as the decision function boundary is swept through the decision space as illustrated in (a) by the green arrow. The inset shows a confusion matrix for the two-class IZA vs. DEEM classification using the full power spectrum SOAP vectors. The superscript † in the confusion matrix labels refers to the predicted labels.

Model Ensemble and the SVM brain

Receiver Operating Characteristic Curves

Fig. S5 provides the receiver operating characteristic (ROC) curves for the full set of "knock-out" models. The worst-performing models are those that rely only on radial (two-body) information on the Si atoms; when three-body correlations and/or correlations involving O atoms are accounted for, the SVM is able to distinguish the IZA frameworks from the DEEM rather well, with areas under the ROC curves (AUCs) improving from 0.86 to 0.93 or higher (Tables S2 and S3). Marginal improvements are observed by increasing the cutoff of the considered correlations from 3.5 Å to 6.0 Å or (in some cases) by considering multiple correlations simultaneously (e.g., accounting for Si-O-Si and Si-Si-Si correlations instead of Si-O-Si or Si-Si-Si correlations alone). Additionally, a model that considers only Si-O-O and Si-Si-Si radial and angular correlations up to 6.0 Å performs *better* than the full model that

includes all Si and O angular correlations. This indicates that the accuracy is data-limited: as long as the set of known zeolites is limited to a few hundred frameworks, it is unlikely that increasing further the complexity of descriptors and models will improve the classification performance.

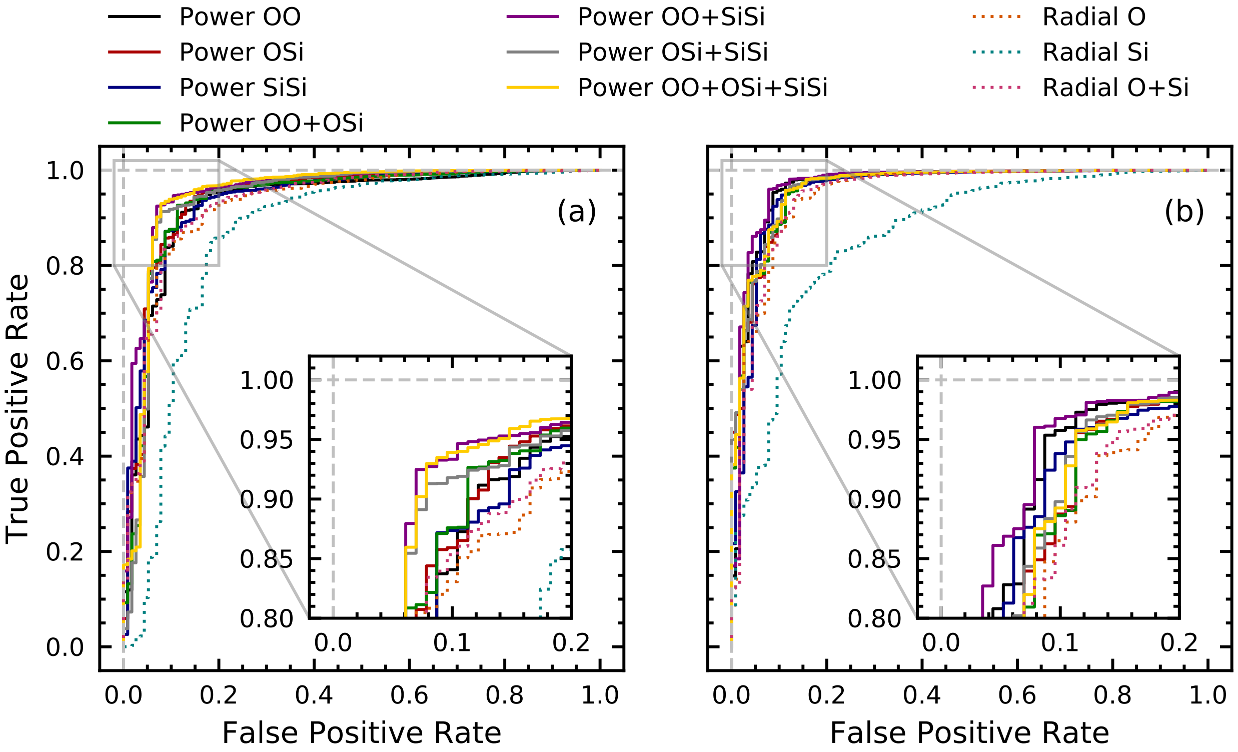

Figure S5: Receiver operator characteristic (ROC) curves for the two-class IZA vs. DEEM SVM classification exercise where subsets of the power spectrum or radial spectrum were used for the SVM training and classification. Panel (a) shows the ROC curves for SVM models based on SOAP features with a 3.5 Å environment cutoff, and Panel (b) shows the ROC curves for SVM models based on SOAP features with a 6.0 Å cutoff. The power spectrum features yield better predictions than the radial spectrum, with Si-O-Si correlations being particularly important for the classification.

Confusion Matrices

Fig. S6 shows the two-class IZA vs. DEEM confusion matrices for the full complement of "knock-out" models. In the two-class case, all of the power spectrum (three-body) models correctly classify approximately 100 of the 115 IZA frameworks in the test set; these models differ mainly in the number of misclassified DEEM frameworks, ranging from approximately

Table S2: AUCs of the two-class IZA vs. DEEM classifications for the ensemble of SVM models based on the SOAP power spectrum

	Power Spectrum						
	OO	OSi	SiSi	OO+OSi	OO+SiSi	OSi+SiSi	OO+OSi+SiSi
3.5 Å	0.931	0.943	0.940	0.937	0.958	0.941	0.949
6.0 Å	0.966	0.964	0.959	0.964	0.970	0.966	0.966

Table S3: AUCs of the two-class IZA vs. DEEM classifications for the ensemble of SVM models based on the radial spectrum

	Radial Spectrum		
	O	Si	O+Si
3.5 Å	0.930	0.862	0.932
6.0 Å	0.948	0.866	0.948

8,000 to 17,000 for the 6.0 Å models and from 19,000 to 30,000 for the 3.5 Å models. The radial spectrum (two-body) models that include information on O correlations also correctly classify approximately 100 IZA frameworks, but tend to misclassify many more DEEM frameworks than the power spectrum models. As noted in the discussion of Fig. S5, the radial spectrum models that account only for Si atom information show the worst performance, correctly classifying less than 100 IZA frameworks and misclassifying more than 60,000 DEEM frameworks.

Fig. S7 similarly shows the four-class IZA1 vs. IZA2 vs. IZA3 vs. DEEM confusion matrices for the full ablation study. The four-class classification exercise is much more difficult than the two-class case, in part due to the fact that many IZA topologies can be synthesized with a variety of different compositions, making it difficult to divide the frameworks into a set of mutually exclusive categories (as a reminder, we have chosen the chemical composition of the first entry listed in the IZA database for each topology). Nevertheless, the SVM is able to distinguish the four classes much better than a random guess, and the resulting predictions provide some insights into the structural characteristics of the different types of IZA frameworks. In general, while the SVM occasionally misclassifies an IZA1 (all-silica)

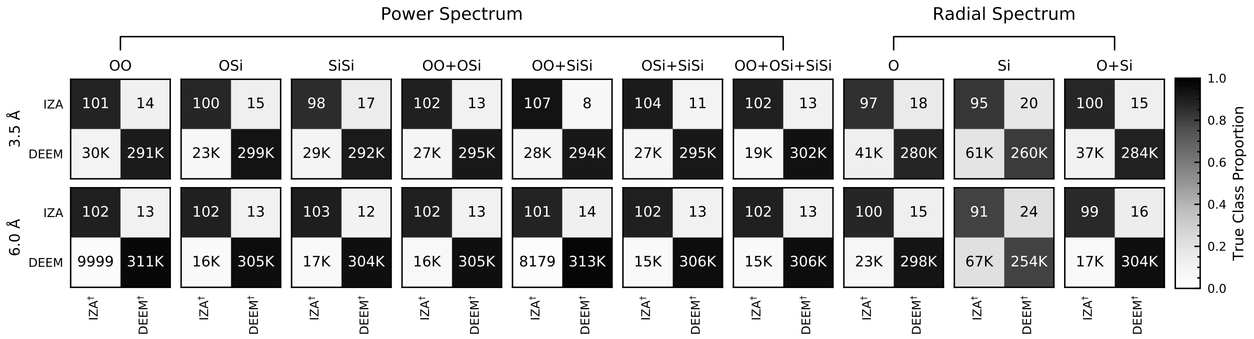

Figure S6: Confusion matrices from the two-class SVM classification where subsets of the power spectrum or radial spectrum were used for the SVM training and classification. The rows of the confusion matrices correspond to the *true* class labels, while the columns correspond to the *predicted* class labels and are denoted by the superscript †. The matrix entries are colored according to the proportion of the true labels that have been predicted as a particular class, while the interior text gives the absolute number of such classifications.

framework as belonging to IZA2 (Si, O, and heteroatoms), rarely does the SVM classify an IZA1 framework as IZA3 (containing O but no Si). Similarly, IZA3 frameworks are often misclassified as IZA2, but are much less often misclassified as IZA1. This suggests that the composition of a framework indeed affects its topology, and that such effects are preserved even upon conversion to an all-silica form.

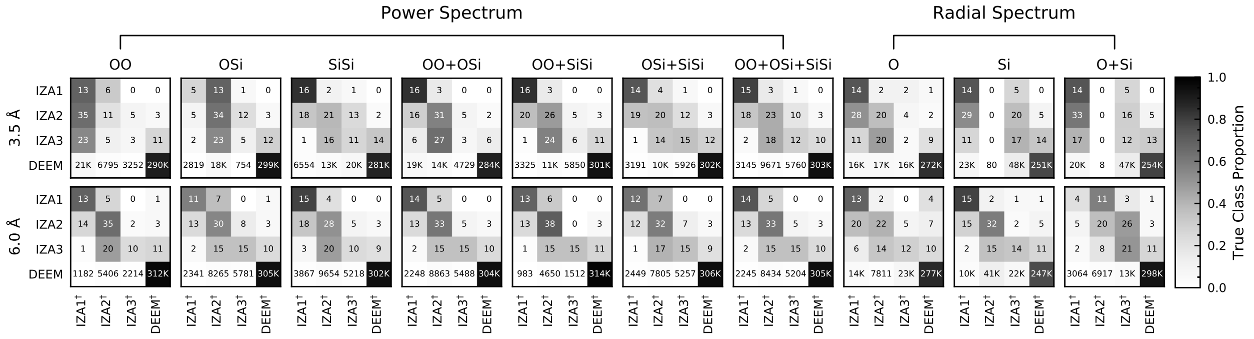

Figure S7: Confusion matrices from the four-class SVM classification where subsets of the power spectrum or radial spectrum were used for the SVM training and classification. The rows of the confusion matrices correspond to the *true* class labels, while the columns correspond to the *predicted* class labels and are denoted by the superscript †. The matrix entries are colored according to the proportion of the true labels that have been predicted as a particular class, while the interior text gives the absolute number of such classifications. While the classifier often correctly classifies the DEEM frameworks as such, it has more difficulty distinguishing between the IZA subcategories.

As a simple check to ascertain whether the SVM classification predictions we observe

are made based on genuine structural differences between the IZA and DEEM frameworks, we also performed two- and four-class classification exercises on a random split of only the DEEM frameworks: the DEEM structures were divided randomly into two (or four) different artificial classes of approximately the same size, and an SVM was trained on the randomly assigned labels for the DEEM frameworks in the training set for each model in the ablation study. In particular, we take the DEEM frameworks from the train and test sets used to build the SVM and PCovR models and assign classes for these frameworks randomly as either 1 or 2 (in the two-class case) or 1 to 4 (in the four-class case) from a discrete uniform distribution, so that the class populations are approximately equal. We then construct the SVM models as described previously. The resulting confusion matrices for predictions of these randomly assigned labels for DEEM frameworks in the test set are provided in Figs. S8 and S9. For every model in both the two- and four-class cases, the SVM is not able to assign class labels more accurately than a random guess. The inability of the SVM to distinguish between the arbitrarily assigned artificial DEEM classifications suggests that the classification predictions we observe in the IZA vs. DEEM exercises are based on genuine structural differences between the IZA and DEEM frameworks in our dataset.

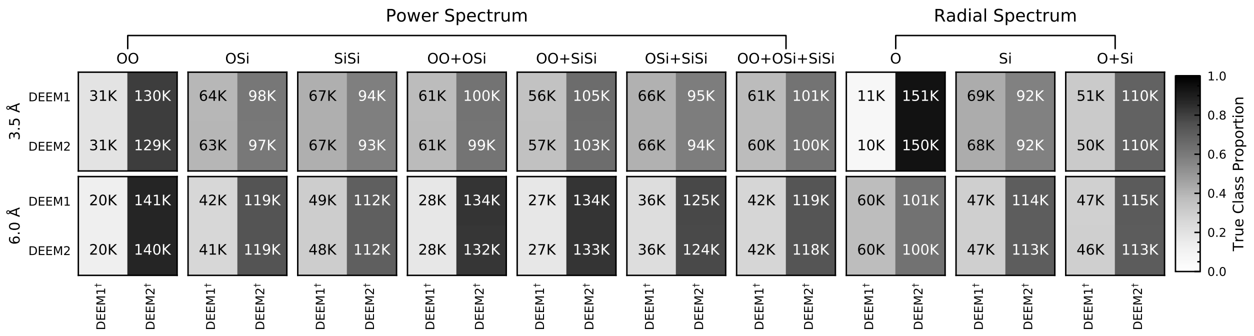

Figure S8: SVM confusion matrices for a random two-class split of a subset of frameworks from the DEEM database. The SVM cannot distinguish between the arbitrarily assigned classes.

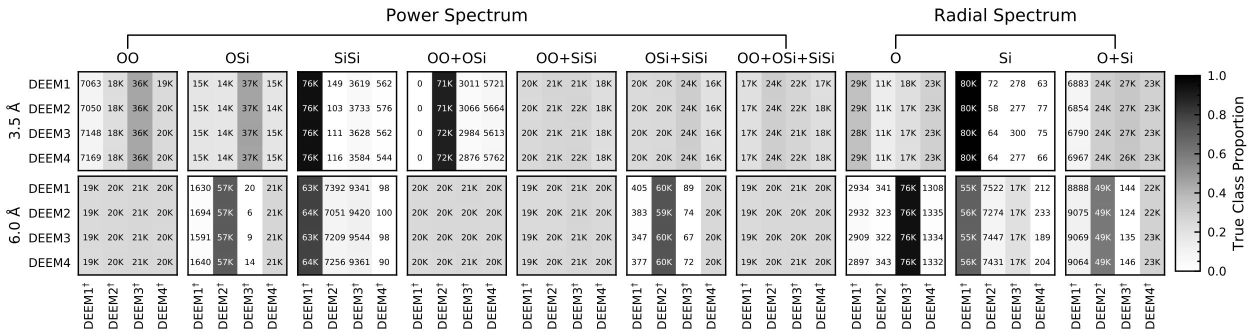

Figure S9: SVM confusion matrices for a random four-class split of a subset of frameworks from the DEEM database. The SVM cannot distinguish between the arbitrarily assigned classes.

SVM "Brains"

Fig. S10 provides the real-space representation of the SVM decision-making process for the 3.5 Å and 6.0 Å radial spectrum models; Fig. S10(e) plots the same data as in Fig. 4(e) of the main text. In each subplot, the faded red and blue lines are the decision traces (see discussion of Fig. 4 (e) in the main text) for the same 25 randomly selected IZA frameworks and 25 randomly selected DEEM frameworks, and the solid black line indicates the class-averaged density for the structures in the training set. In both the 3.5 Å and 6.0 Å models, structural features around ≈ 3 Å appear the most influential in the SVM decision-making process, as this is the region that exhibits the largest collective changes in the decision trace. In the 6.0 Å models, structural correlations past 3.5 Å can also substantially impact the final decision, particularly in the models accounting for only Si-O or Si-Si correlations (Figs. S10(e) and (f)). Furthermore, in models that account for both Si-O and Si-Si correlations, the Si-Si correlations appear to have minimal impact on the final classification. This is evident from Figs. S10(c) and (d) for the 3.5 Å model and from Figs. S10(g) and (h) for the 6.0 Å model, which plot separately the contributions from Si-O correlations and Si-Si correlations in an SVM model that accounts for both sets of correlations in making classification decisions. The decision traces of the Si-Si correlations are rather flat and sign changes are rare, suggesting that such structural information has only a minimal impact on the final class assignment.

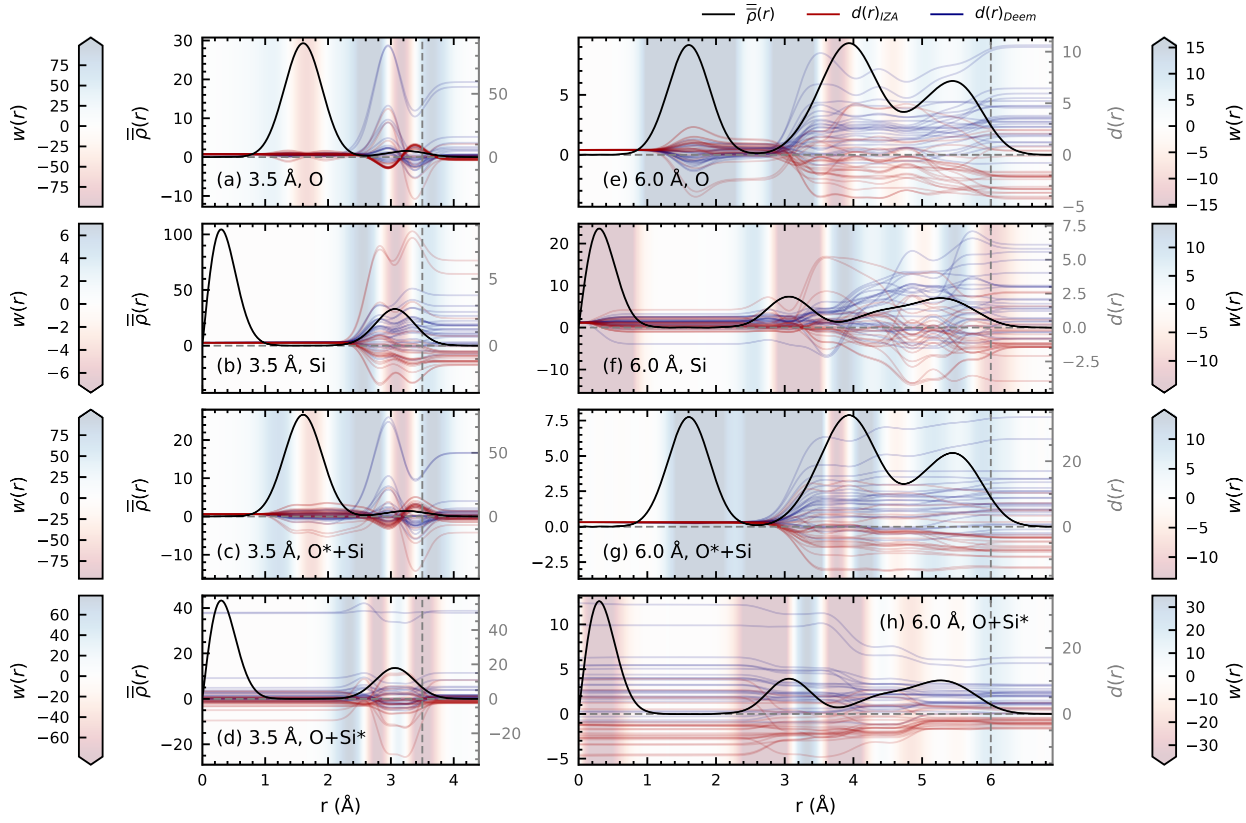

Figure S10: Decision traces $d(r)$ for 25 random IZA and 25 random DEEM frameworks and the class-averaged radial density $\bar{\bar{\rho}}(r)$ reconstructed from the radial spectrum SOAP features for frameworks in the train set. The plot background is colored according the the value of the SVM weights $w(r)$, and is truncated to better show sign changes: weights falling outside the colorbar limits are colored corresponding to the appropriate end of the colorbar. The SVM decision boundary $d(r) = 0$ is given by the horizontal dashed line; the SOAP environment cutoff is given by the vertical dashed line. All subplots are labeled with the model cutoff and the particular correlations used to train the corresponding SVM. The labels "Si+O*" and "Si*+O" are used to indicate that the SVM was trained on both Si-Si and Si-O correlations, but the plot shows only the decision traces for the Si-O or Si-Si correlations, respectively. In these cases, the decision traces for the Si-Si correlation plots (labeled "Si*+O", subplots (d) and (h)) do not begin at zero, but rather at the $r \to \infty$ limit of the Si-O correlations (labeled "Si+O*", subplots (c) and (g)), with the $r \to \infty$ limit of the Si-Si correlations providing the final decision function values for the SVM models trained on both Si-Si and Si-O correlations. This particular arrangement allows one to compare the relative importance in the species-wise correlations in the SVM decision-making process.

PCovR setup

In order to solve class imbalance for the dataset, we chose to replicate samples from the minority class to achieve approximate class parity rather than a minority oversampling technique like SMOTE,[S8] as this would introduce hypothetical samples into the set of IZA frameworks, distorting our baseline that the IZA class contains only structures that are known to be experimentally synthesizable. The preprocessing of the feature and target data is performed on the replicated data and in the same way as in the SVM case. It should be noted that the target data in the multi-class case (the multi-class SVM decision functions), are scaled globally (i.e., by the Frobenius norm) rather than columnwise; they are still centered columnwise. The PCovR mixing and regression regularization are optimized using a two-fold cross-validation scheme. The optimal regularization is determined through an order-of-magnitude grid search with a search range of 10^{-10} to 10^{0}, and the optimal mixing is similarly determined through a grid search with the set $\{0.0, 0.1, 0.2, \ldots 0.8, 0.9, 1.0\}$.

Candidate Selection

Fig. S11 shows a schematic of the workflow used to identify the synthesis candidates from the database of DEEM frameworks. The resulting hierarchy of candidates is given in Table S4, which show the five DEEM frameworks in the 55–60 Å^3/Si, 60–65 Å^3/Si, and 65–70 Å^3/Si volume categories ordered by their distance to the convex hull along the energy direction. The database ID of the framework in each volume category that is closest to the hull is given in bold. These highlighted frameworks are shown in Fig. S12 alongside the closest IZA framework in the full 6.0 Å power spectrum SOAP space. All three DEEM candidates appear similar to their IZA counterparts along one axis, but are noticeably different upon further inspection. The full collection of candidate structures can be examined with the `chemiscope` viewer[S9] at the following link:

https://chemiscope.org/?load=https://www.epfl.ch/labs/cosmo/wp-content/uploads/2021/10/zeolite_sorting_hat-selected.chemiscope.json_.gz

(Some PDF viewers require you to click on the second line of the link above. If necessary, please copy the link above and paste it into your browser.) `chemiscope` is a visualization utility that facilitates the exploration of materials datasets by highlighting structure–property relationships, allowing users to construct "maps" of materials based on their properties. Within the map, a user can select a particular point and obtain information about the corresponding atomic structure (or environment).

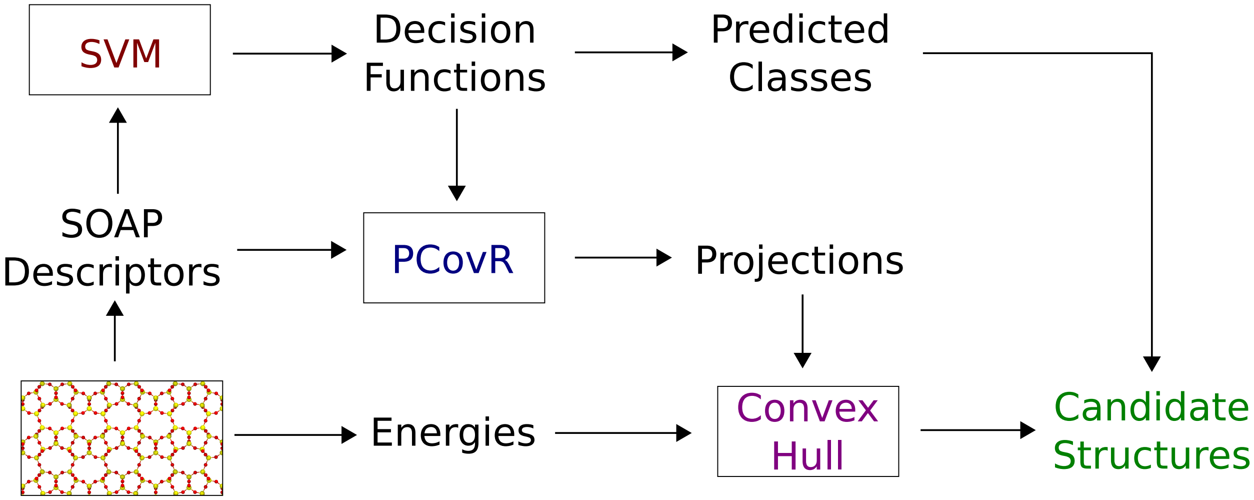

Figure S11: Schematic of the SVM-PCovR-GCH infrastructure. The GULP energies and SOAP descriptors are computed for each framework, and the SOAP descriptors are used as input to both SVM and PCovR models. The decision functions resulting from the SVM classification are additionally used as input to the PCovR model, where they are combined with the SOAP features to develop a latent space projection that serves as the basis for a GCH construction using the GULP energies as a measure of thermodynamic stability. The structures near the convex hull can then be compared against the SVM classification predictions to create a hierarchy of synthesis candidates.

Table S4: Candidate DEEM frameworks having molar volumes between 55–60 Å^3/Si, 60–65 Å^3/Si, and 65–70 Å^3/Si. The five candidates closest to the convex hull in each volume category are listed. For each DEEM candidate, its ID, molar volume V (units Å^3/Si), predicted silicon fraction f_{DEEM}, distance from the convex hull along the energy axis E_{hull} (units kJ/mol Si), and two-class decision function value d are listed, alongside the IZA code, true Si fraction f_{IZA}, and true house designation of the three nearest IZA frameworks in SOAP space with corresponding distance d_{IZA}. IDs listed in bold correspond to those shown in Fig. S12.

Volume Group	Group Rank	ID	V	f_{DEEM}	E_{hull}	F	Predicted House	IZA Code	f_{IZA}	IZA House	d_{IZA}
								THO	0.17	IZA2	4.40×10^{-4}
	(1)	**8158735**	59.51	0.11	-2.02×10^{-12}	-2.14	IZA2	EDI	0.20	IZA2	4.44×10^{-4}
								LOV	0.26	IZA2	5.04×10^{-4}
								TOL	0.17	IZA2	2.91×10^{-4}
	(2)	8322701	56.01	0.15	1.56×10^{-1}	-1.25	IZA3	FRA	0.17	IZA2	3.32×10^{-4}
								FAR	0.17	IZA2	3.80×10^{-4}
								TOL	0.17	IZA2	2.59×10^{-4}
55–60 Å^3	(3)	8322800	55.96	0.14	3.43×10^{-1}	-1.28	IZA3	LTN	0.17	IZA2	3.71×10^{-4}
								FRA	0.17	IZA2	3.83×10^{-4}
								FRA	0.17	IZA2	4.89×10^{-5}
	(4)	8327193	57.07	0.17	3.69×10^{-1}	-1.19	IZA2	FAR	0.17	IZA2	1.57×10^{-4}
								MAR	0.17	IZA2	2.00×10^{-4}
								TOL	0.17	IZA2	2.37×10^{-4}
	(5)	8322704	55.92	0.14	4.28×10^{-1}	-1.22	IZA3	LTN	0.17	IZA2	3.75×10^{-4}
								GIU	0.17	IZA2	3.90×10^{-4}
								SBN	0.00	IZA3	2.41×10^{-4}
	(1)	**8054476**	61.06	0.14	-2.71×10^{-12}	-3.55	IZA2				

								NAB	0.27	IZA2	4.74×10^{-4}
								THO	0.17	IZA2	5.12×10^{-4}
								NAT	0.20	IZA2	7.01×10^{-5}
	(2)	8214845	60.07	0.17	9.81×10^{-2}	-3.20	IZA2	THO	0.17	IZA2	1.41×10^{-4}
								EDI	0.20	IZA2	3.59×10^{-4}
								AFT	0.00	IZA3	1.40×10^{-5}
	(3)	8326505	64.11	0.17	9.84×10^{-1}	-1.74	IZA2	CHA	0.22	IZA2	4.20×10^{-5}
								AFX	0.03	IZA2	4.26×10^{-5}
								ERI	0.25	IZA2	1.64×10^{-4}
	(4)	8325527	60.63	0.16	1.14	-1.79	IZA2	LEV	0.22	IZA2	1.67×10^{-4}
								SWY	0.03	IZA2	1.74×10^{-4}
								AFT	0.00	IZA3	1.07×10^{-5}
	(5)	8323843	64.09	0.17	1.20	-1.67	IZA2	AFX	0.03	IZA2	1.81×10^{-5}
								SFW	0.30	IZA2	4.54×10^{-5}
								RHO	0.25	IZA2	1.34×10^{-4}
	(1)	**8312395**	66.17	0.17	3.04	-1.66	IZA3	PWN	0.26	IZA2	2.30×10^{-4}
								PAU	0.26	IZA2	3.20×10^{-4}
								RHO	0.25	IZA2	1.06×10^{-4}
	(2)	8315456	65.14	0.18	3.12	-1.65	IZA3	PWN	0.26	IZA2	2.09×10^{-4}
								PAU	0.26	IZA2	3.02×10^{-4}
								EMT	0.26	IZA2	1.60×10^{-4}
65–70 Å^3	(3)	8321687	67.68	0.14	3.98	-1.63	IZA3	FAU	0.23	IZA2	1.71×10^{-4}

							TSC	0.17	IZA2	2.31×10^{-4}
							PWN	0.26	IZA2	2.81×10^{-4}
(4)	8308137	65.01	0.16	4.01	-1.62	IZA3	EAB	0.25	IZA2	2.87×10^{-4}
							AVL	0.00	IZA3	2.94×10^{-4}
							LTA	0.17	IZA2	1.80×10^{-4}
(5)	8314368	65.92	0.12	4.16	-2.81	IZA3	IRN	0.27	IZA2	3.23×10^{-4}
							UFI	0.29	IZA2	3.48×10^{-4}

Computational tools

The machine learning analyses were performed in Python[S10,S11] with generous use of the `numpy`,[S12–S14] `scipy`,[S15] `scikit-learn`,[S7] `chemiscope`,[S9] and `ase`[S16] packages. Plots were generated with `matplotlib`[S17] and `plotly`,[S18] and atomic snapshots were generated with VESTA.[S19]

References

(S1) Sanders, M. J.; Leslie, M.; Catlow, C. R. A. Interatomic potentials for SiO2. *J. Chem. Soc., Chem. Commun.* **1984**, 1271.

(S2) Pophale, R.; Cheeseman, P. A.; Deem, M. W. A Database of New Zeolite-Like Materials. *Physical Chemistry Chemical Physics* **2011**, *13*, 12407–12412.

(S3) Goscinski, A.; Musil, F.; Pozdnyakov, S.; Nigam, J.; Ceriotti, M. Optimal Radial Basis for Density-Based Atomic Representations. *J. Chem. Phys.* **2021**, *155*, 104106.

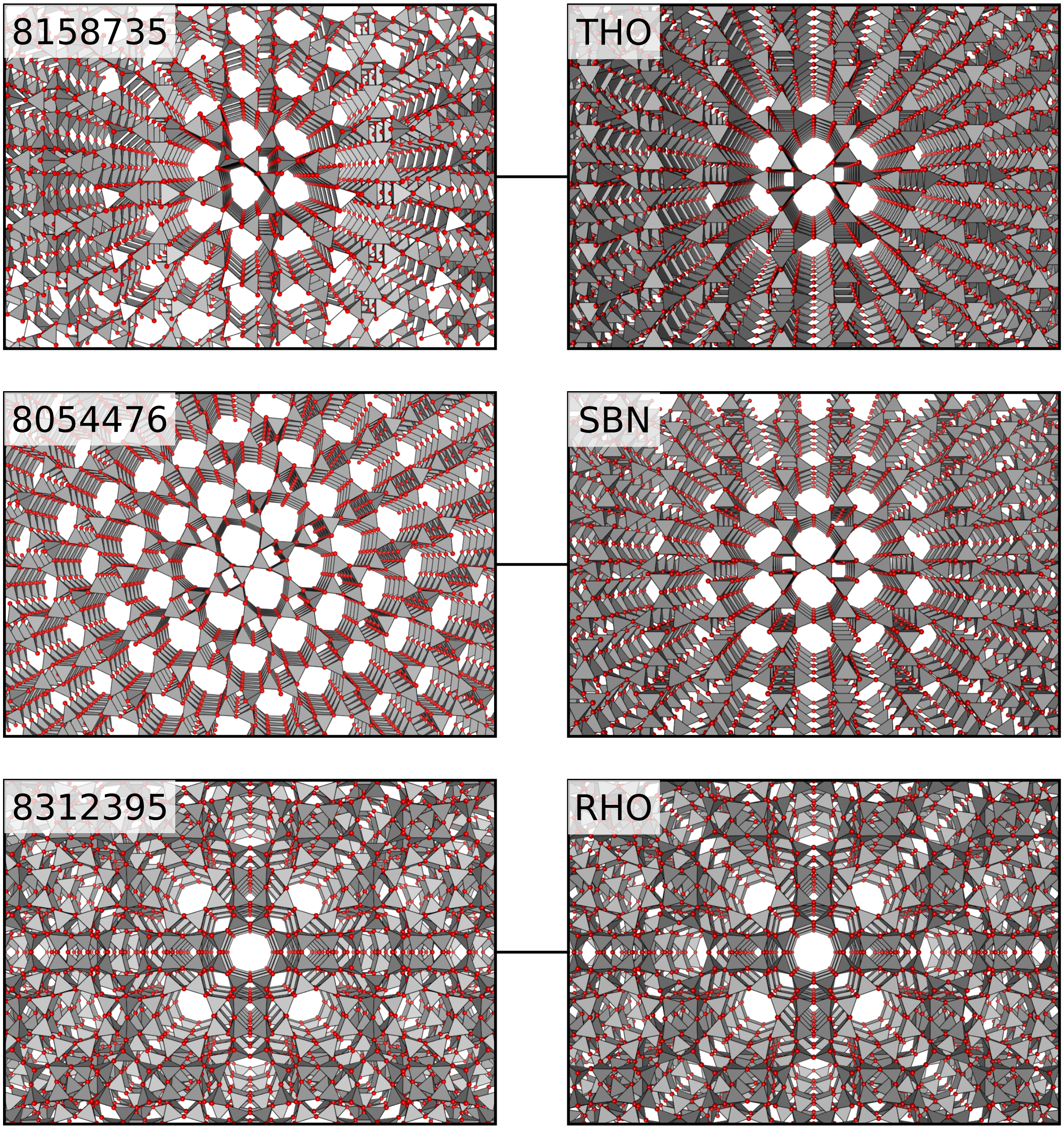

Figure S12: DEEM candidates for the three volume groups and their nearest-neighbor IZA framework in the full 6.0 Å power spectrum SOAP space.

(S4) Helfrecht, B. A.; Semino, R.; Pireddu, G.; Auerbach, S. M.; Ceriotti, M. A new kind of atlas of zeolite building blocks. *The Journal of Chemical Physics* **2019**, *151*, 154112.

(S5) Anelli, A.; Engel, E. A.; Pickard, C. J.; Ceriotti, M. Generalized Convex Hull Construction for Materials Discovery. *Phys. Rev. Mater.* **2018**, *2*, 103804.

(S6) Musil, F.; Veit, M.; Junge, T.; Stricker, M.; Goscinki, A.; Fraux, G.; Ceriotti, M. LIBRASCAL. `https://github.com/cosmo-epfl/librascal`, 2020.

(S7) Pedregosa, F.; Varoquaux, G.; Gramfort, A.; Michel, V.; Thirion, B.; Grisel, O.; Blondel, M.; Prettenhofer, P.; Weiss, R.; Dubourg, V.; Vanderplas, J.; Passos, A.; Cournapeau, D.; Brucher, M.; Perrot, M.; Duchesnay, E. Scikit-Learn: Machine Learning in Python. *Journal of Machine Learning Research* **2011**, *12*, 2825–2830.

(S8) Chawla, N. V.; Bowyer, K. W.; Hall, L. O.; Kegelmeyer, W. P. SMOTE: Synthetic Minority Over-Sampling Technique. *Journal of Artificial Intelligence Research* **2002**, *16*, 321–357.

(S9) Fraux, G.; Cersonsky, R. K.; Ceriotti, M. Chemiscope: Interactive Structure-Property Explorer for Materials and Molecules. *Journal of Open Source Software* **2020**, *5*, 2117.

(S10) Millman, K. J.; Aivazis, M. Python for Scientists and Engineers. *Computing in Science & Engineering* **2011**, *13*, 9–12.

(S11) Oliphant, T. E. Python for Scientific Computing. *Computing in Science Engineering* **2007**, *9*, 10–20.

(S12) Oliphant, T. E. *Guide to NumPy*, 2nd ed.; CreateSpace Independent Publishing Platform, 2015.

(S13) van der Walt, S.; Colbert, S. C.; Varoquaux, G. The NumPy Array: A Structure for Efficient Numerical Computation. *Computing in Science Engineering* **2011**, *13*, 22–30.

(S14) Harris, C. R.; Millman, K. J.; van der Walt, S. J.; Gommers, R.; Virtanen, P.; Cournapeau, D.; Wieser, E.; Taylor, J.; Berg, S.; Smith, N. J.; Kern, R.; Picus, M.; Hoyer, S.; van Kerkwijk, M. H.; Brett, M.; Haldane, A.; del Río, J. F.; Wiebe, M.; Peterson, P.; Gérard-Marchant, P.; Sheppard, K.; Reddy, T.; Weckesser, W.; Abbasi, H.; Gohlke, C.; Oliphant, T. E. Array Programming with NumPy. *Nature* **2020**, *585*, 357–362.

(S15) Virtanen, P.; Gommers, R.; Oliphant, T. E.; Haberland, M.; Reddy, T.; Cournapeau, D.; Burovski, E.; Peterson, P.; Weckesser, W.; Bright, J.; van der Walt, S. J.; Brett, M.; Wilson, J.; Millman, K. J.; Mayorov, N.; Nelson, A. R. J.; Jones, E.; Kern, R.; Larson, E.; Carey, C. J.; Polat, I.; Feng, Y.; Moore, E. W.; VanderPlas, J.; Laxalde, D.; Perktold, J.; Cimrman, R.; Henriksen, I.; Quintero, E. A.; Harris, C. R.; Archibald, A. M.; Ribeiro, A. H.; Pedregosa, F.; van Mulbregt, P. SciPy 1.0: Fundamental Algorithms for Scientific Computing in Python. *Nature Methods* **2020**, *17*, 261–272.

(S16) Hjorth Larsen, A.; Jø rgen Mortensen, J.; Blomqvist, J.; Castelli, I. E.; Christensen, R.; Duł ak, M.; Friis, J.; Groves, M. N.; Hammer, B. r.; Hargus, C.; Hermes, E. D.; Jennings, P. C.; Bjerre Jensen, P.; Kermode, J.; Kitchin, J. R.; Leonhard Kolsbjerg, E.; Kubal, J.; Kaasbjerg, K.; Lysgaard, S.; Bergmann Maronsson, J.; Maxson, T.; Olsen, T.; Pastewka, L.; Peterson, A.; Rostgaard, C.; Schiø tz, J.; Schütt, O.; Strange, M.; Thygesen, K. S.; Vegge, T.; Vilhelmsen, L.; Walter, M.; Zeng, Z.; Jacobsen, K. W. The Atomic Simulation Environment—a Python Library for Working with Atoms. *Journal of Physics: Condensed Matter* **2017**, *29*, 273002.

(S17) Hunter, J. D. Matplotlib: A 2D Graphics Environment. *Computing in Science Engineering* **2007**, *9*, 90–95.

(S18) Plotly Technologies Inc., Collaborative data science. 2015; `https://plot.ly`.

(S19) Momma, K.; Izumi, F. VESTA 3 for Three-Dimensional Visualization of Crystal,

Volumetric and Morphology Data. *Journal of Applied Crystallography* **2011**, *44*, 1272–1276.
